# Preparation of nickel-iron hydroxides by microorganism corrosion for efficient oxygen evolution

**DOI:** 10.1038/s41467-020-18891-x

**Published:** 2020-10-08

**Authors:** Huan Yang, Lanqian Gong, Hongming Wang, Chungli Dong, Junlei Wang, Kai Qi, Hongfang Liu, Xingpeng Guo, Bao Yu Xia

**Affiliations:** 1 Key Laboratory of Material Chemistry for Energy Conversion and Storage (Ministry of Education), Hubei Key Laboratory of Material Chemistry and Service Failure, Wuhan National Laboratory for Optoelectronics, School of Chemistry and Chemical Engineering, Huazhong University of Science and Technology (HUST), 1037 Luoyu Road, Wuhan, 430074 China; 2 Institute for Advanced Study, Nanchang University, 999 Xuefu Road, Nanchang, China; 3 Department of Physics, Tamkang University, 151 Yingzhuan Road, New Taipei City, 25137 Taiwan

**Keywords:** Electrocatalysis, Corrosion, Energy, Corrosion, Electrocatalysis

## Abstract

Nickel–iron composites are efficient in catalyzing oxygen evolution. Here, we develop a microorganism corrosion approach to construct nickel–iron hydroxides. The anaerobic sulfate-reducing bacteria, using sulfate as the electron acceptor, play a significant role in the formation of iron sulfide decorated nickel–iron hydroxides, which exhibit excellent electrocatalytic performance for oxygen evolution. Experimental and theoretical investigations suggest that the synergistic effect between oxyhydroxides and sulfide species accounts for the high activity. This microorganism corrosion strategy not only provides efficient candidate electrocatalysts but also bridges traditional corrosion engineering and emerging electrochemical energy technologies.

## Abstract (web summary)

Developing facile strategies to realize the precise construction of Ni-Fe structures is of significance for water oxidation. Here, the authors demonstrate a universal microorganism-assisted corrosion strategy for preparing highly efficient Ni-Fe composites towards oxygen evolution.

## Introduction

Oxygen evolution is of great significance in several energy conversion systems including rechargeable metal-air batteries and water electrolysis devices^1–4^. However, the sluggish kinetics in the complicated multiple proton/electron-processes requires highly efficient electrocatalysts^5,6^. Noble metal (Ru, Ir, etc.) based nanocomposites manifest high activities, but the limited earth reserves and high cost cannot support their practical applications. Numerous earth-abundant alternatives including metal (hydro)oxides and sulfides have been developed to replace precious electrocatalysts^7–15^. Among them, Ni–Fe oxides/hydroxides have been demonstrated as the excellent electrocatalysts for oxygen evolution in alkaline electrolytes^15–17^.

Various methods including electrodeposition and hydrothermal treatment have been developed to prepare Ni–Fe oxides/hydroxides^18,19^. These bottom-up methods require the meticulous treatment of the complex precursor at stringent synthetic conditions (high temperature, voltage or pressure) to realize the precise construction of nanostructures^20–23^. Generally, metal etching is a common phenomenon occurred at ambient temperature and pressure in corrosion engineering. Thus, this top–down approach by etching metallic substrates provides new opportunities to build the integrated electrodes at mild environment^24–27^. Moreover, natural microbial usually promote the corrosion behaviors and bring the incorporation of heteroatom species. For example, the main corrosion products on carbon steel are iron sulfides and iron (hydroxy)oxides in the presence of sulfate reducing bacteria (SRB)^28^. These corrosion products containing metal sulfides/(hydro)oxides have potential activity for oxygen evolution^29–31^. Nevertheless, few studies have focused on this natural microorganism corrosion induced biofilm electrodes.

Inspired by the natural microorganism-assisted corrosion behaviors occurred on pipeline and equipment in oil fields, here we demonstrate the preparation of highly active Ni–Fe composites by corrosion engineering in the presence of anaerobic SRB. The as-prepared electrode exhibits excellent activity for oxygen evolution with an overpotential of only 220 mV needed to achieve the benchmark current density of 10 mA cm^−2^. Experimental and theoretical studies suggest that the synergistic effect between oxyhydroxide from chemical corrosion and iron sulfide species from microorganism corrosion would account for the high activity. This work not only provides an efficient alternative electrocatalyst but also more importantly introduces a facile corrosion strategy, which would attract broad interest from multidisciplinary fields of natural biology, traditional metal corrosion and modern electrochemical energy technologies.

## Results

### Formation and structural characterization of corrosion electrodes

**Fig. 1:**
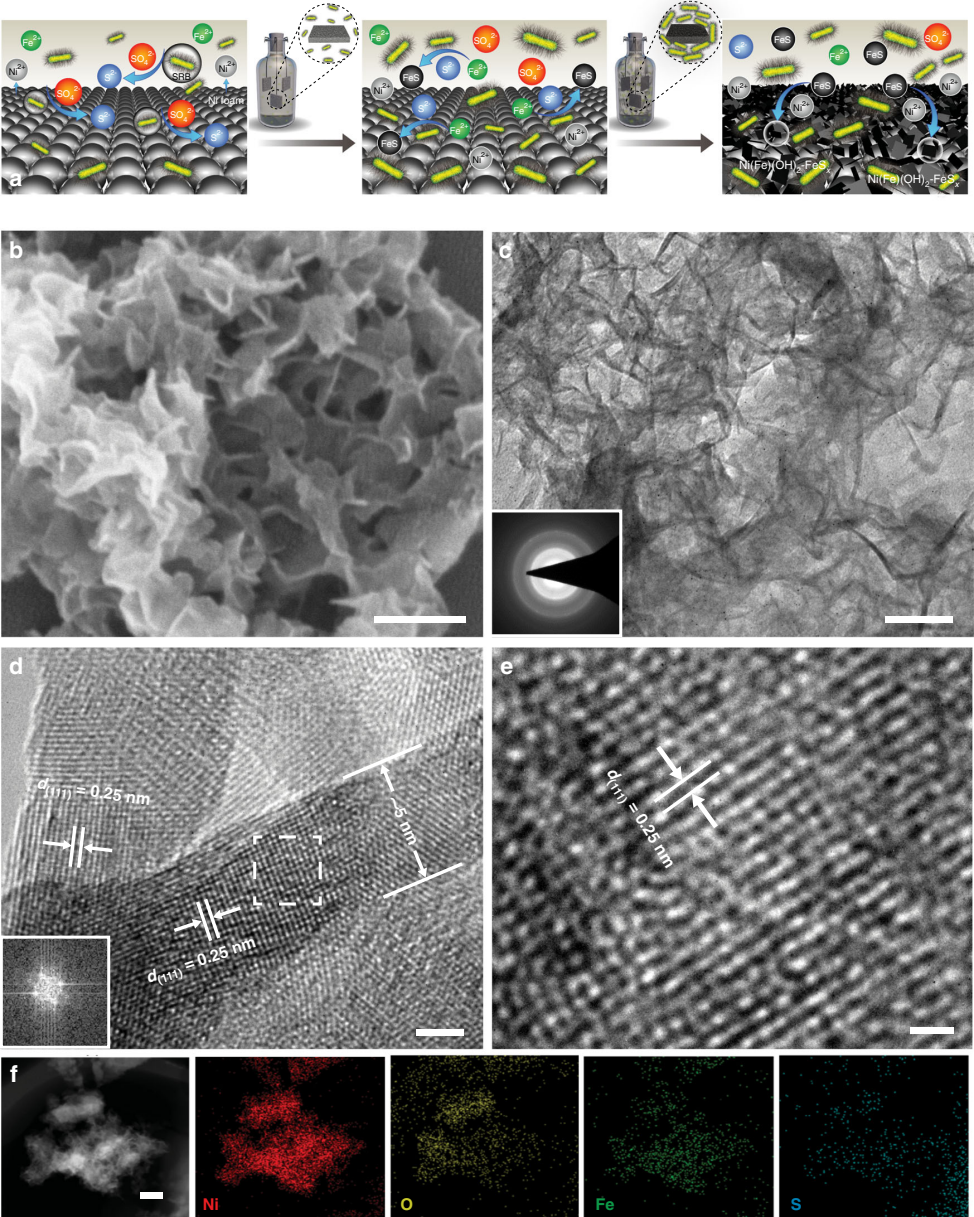
Formation and structural characterizations of Ni(Fe)(OH)_2_–FeS_*x*_. **a** Preparation scheme of the SRB-assisted corrosion-formed electrode. **b** FESEM image, scale bar: 200 nm. **c** TEM image and corresponding SAED pattern (inset), scale bar: 50 nm. **d**, **e** HRTEM images and corresponding fast Fourier transform (FFT) pattern (inset in **d**), scale bars: 2 nm (**d**) and 1 nm (**e**). **f** corresponding elemental mappings, scale bar: 250 nm.

**Fig. 2:**
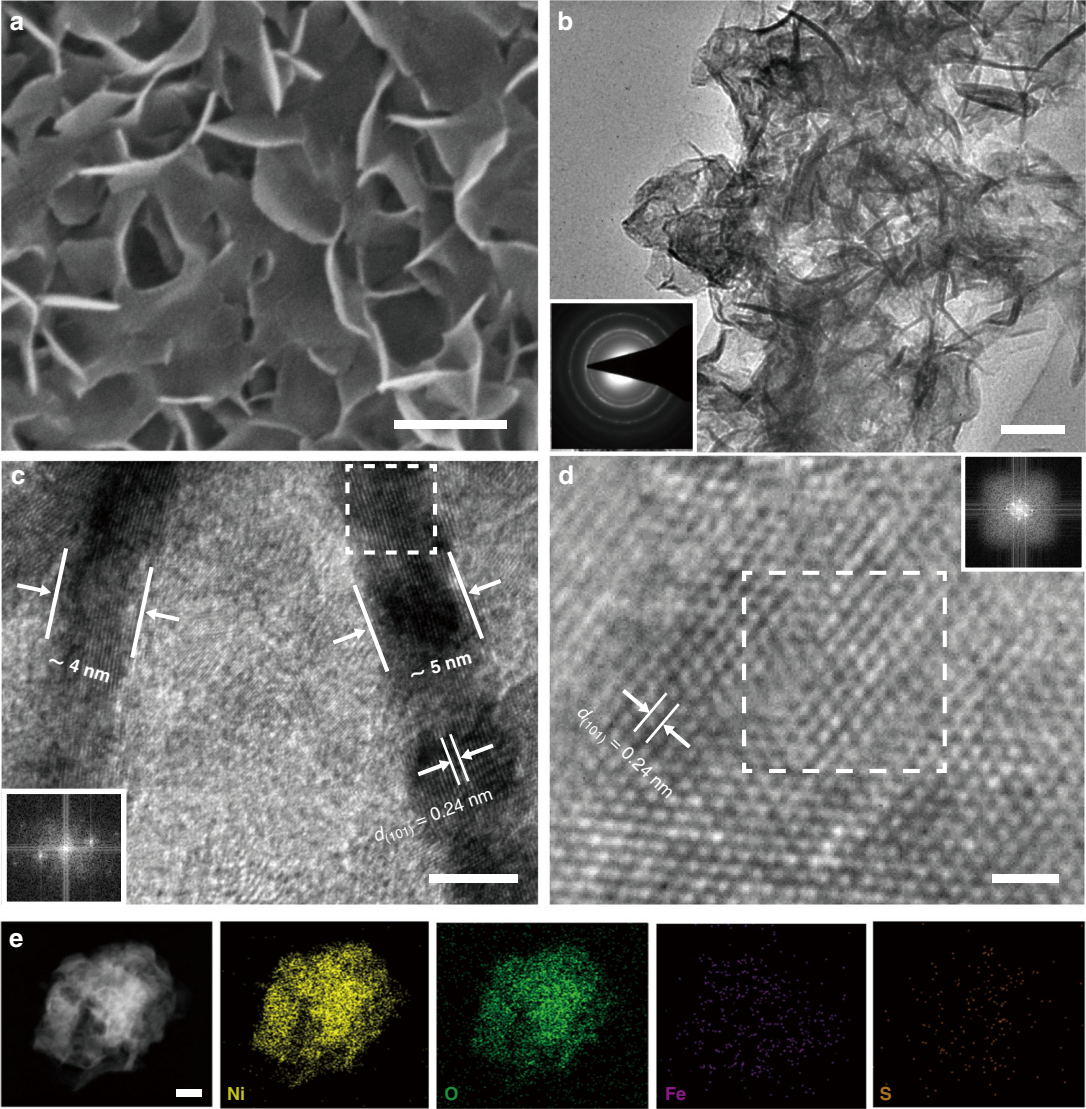
Structural characterizations of Ni(Fe)OOH–FeS_x_. **a** FESEM image, scale bar: 200 nm. **b** TEM image and corresponding SAED pattern (inset), scale bar: 50 nm. **c**, **d** HRTEM images and corresponding FFT patterns (insets), scale bars: 5 nm (**c**) and 1 nm (**d**). **e** corresponding elemental mappings, scale bar: 50 nm.

Fig. 1a illustrates the formation process of iron sulfides decorated nickel–iron hydroxide (Ni(Fe)(OH)_2_–FeS_*x*_) biofilm in the SRB-involved corrosion system. The color of Ni foam turns gradually from gray to black with the increase of corrosion time in the SRB solution, and the corrosion electrodes can be prepared with different scales (Supplementary Fig. 1). Generally, SRB consume the atomic hydrogen absorbed (H_ads_) at the cathode and reduce sulfates to sulfides. Finally, the reaction between sulfides from SRB metabolism and the dissolved nickel species simultaneously leads to the formation of Ni(Fe)(OH)_2_–FeS_*x*_. The corrosion mechanisms are described as follows: the dissolution of Ni foam (Ni → Ni^2+^ + 2e^−^) and the process of SRB metabolism (2H_2_O + 2e^−^ → 2H_ads_ + 2OH^−^; SO_4_^2−^ + 8H_ads_ → S^2−^ + 4H_2_O; Fe^2+^ + S^2−^ → FeS), and then the formation of final SRB corrosion-formed biofilm on the Ni foam (Ni^2+^ + Fe^2+^ + 2OH^−^ + FeS → Ni(Fe)(OH)_2_–FeS_*x*_). Field-emission scanning electron microscopy (FESEM) image reveals the uniform nanosheet arrays deposited onto the skeleton of Ni foam (Fig. 1b). Transmission electron microscopy (TEM) image of the corrosion biofilm shows a highly rippled nanosheet structure (Fig. 1c). High-resolution (HR)TEM images indicate an average thickness of ~5 nm for these nanosheets with an interplanar spacing of 0.25 nm, corresponding to the (111) plane of *α*-Ni(OH)_2_ phase (Fig. 1d, e). These morphology and microstructure features of the Ni(Fe)(OH)_2_–FeS_*x*_ corrosion biofilm are similar to the corrosion products of Ni(Fe)(OH)_2_ obtained in the absence of SRB (Supplementary Fig. 2). Compared with the selected-area electron diffraction (SAED) pattern of Ni(Fe)(OH)_2_ (Supplementary Fig. 2b), more distinct diffraction rings suggest the enhanced crystallinity of Ni(Fe)(OH)_2_–FeS_*x*_ with the incorporation of Fe–S species (Fig. 1c). The elemental mappings show that Fe and S species are homogeneously distributed on the surface of Ni(Fe)(OH)_2_–FeS_*x*_ product (Fig. 1f). The atomic ratio of Ni, O, Fe and S elements is close to 32:64:2:1 (Supplementary Fig. 3). While only Ni, O and Fe elements appear evenly in the Ni(Fe)(OH)_2_ nanosheets (Supplementary Fig. 2d), indicating that S element is not doped into the chemical corrosion product. A short electrochemical activation process promotes the transformation of hydroxides to oxyhydroxides. SEM and TEM images show that the Ni(Fe)OOH–FeS_*x*_ electrode remains the uniform nanosheet arrays (Fig. 2a, b). HRTEM images display the interplanar spacing of 0.24 nm corresponded to the (101) plane of *α*-NiOOH phase (Fig. 2c, d), which suggests the conversion of *α*-Ni(Fe)(OH)_2_ to *α*-Ni(Fe)OOH. The elemental distribution verifies that Fe and S elements still exist on the surface of corrosion-formed biofilm (Fig. 2e). Combined with the X-ray diffraction (XRD) analysis (Supplementary Fig. 4), the diffraction peaks at 33° and 46° for Ni(Fe)(OH)_2_–FeS_*x*_ are related to *α*-Ni(OH)_2_ (JCPDS: 38-0715), while the peaks at 28° and 58° for Ni(Fe)OOH–FeS_*x*_ are corresponding to *α*-NiOOH (JCPDS: 27-0956). These results are consistent with the structural evolution by TEM observations (Figs. 1 and 2). Furthermore, more distinct SAED rings (Fig. 2b inset) and higher XRD intensity of Ni(Fe)OOH–FeS_*x*_ suggest the improved crystallinity of corrosion product after the activation.

### Compositional analysis of corrosion products

**Fig. 3:**
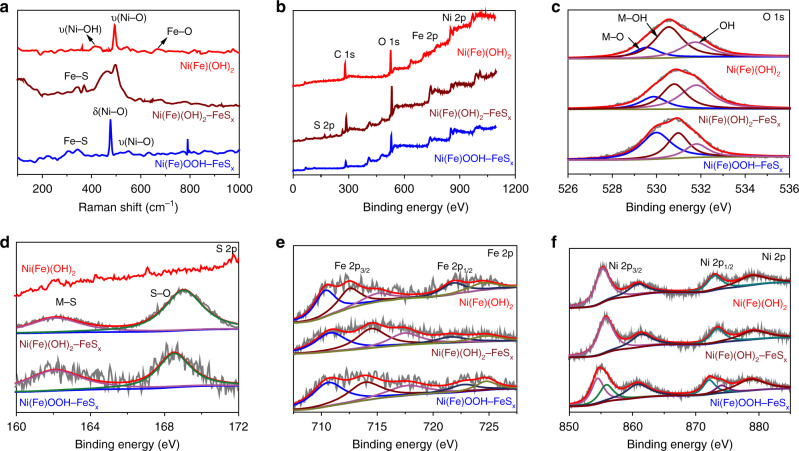
Compositional characterizations of different corrosion-formed products. **a** Raman spectra, **b** XPS surveys, **c**–**f** high-resolution XPS spectra of O 1*s* (**c**), S 2*p* (**d**), Fe 2*p* (**e**) and Ni 2*p* (**f**).

The formation of Fe–S species in SRB-corrosion products is then verified by Raman spectra at 310 and 358 cm^−1^, which are assigned to the typical mackinawite FeS phase (Fig. 3a)^32^. To distinguish the respective effects of microbial metabolism and chemical corrosion on the corroded Ni foam, the presence of Raman peaks at 280–310 and 350–370 cm^−1^ also verify the formation of Fe–S species on the surface of Ti substrates treated in the SRB medium (Supplementary Fig. 5). Moreover, the peaks at 460, 529 and 690 cm^−1^ are respectively attributed to the A_lg_ stretching mode of Ni–OH, Ni–O and Fe–O in the Ni(Fe)(OH)_2_–FeS_*x*_ (Fig. 3a)^18^. After a short activation process, Raman peaks at 479 and 560 cm^−1^ match well with the e_g_ bending vibration and A_1g_ stretching vibration of Ni–O in the Ni(Fe)OOH–FeS_*x*_, and the FeS phase is still preserved (Fig. 3a)^33^. However, the Ni–O peaks are sharpened, and the vibrations assigned to Fe–S in the Ni(Fe)OOH–FeS_*x*_ are significantly shifted and broadened (Fig. 3a), indicating the enhanced crystallinity of corrosion product after the activation, which is consistent with the analysis of XRD and TEM (Fig. 2, Supplementary Fig. 4). The surface composition of corrosion product is further investigated by X-ray photoelectron spectroscopy (XPS). It is confirmed that the Fe–S species only exists in the SRB-involved corrosion product (Fig. 3b, Supplementary Fig. 5c). The deconvoluted O 1*s* spectrum can be fitted into M (Ni/Fe)-O, M-O-H and H_2_O (Fig. 3c, Supplementary Fig. 5d). In the S 2*p* spectrum, two peaks at 162.1 and 168.4 eV are indexed to the M–S and S–O, respectively (Fig. 3d). The S 2*p* peak only exists in the SRB-corrosion products (Supplementary Fig. 5e). Fe 2p regions can be fitted into two pairs of characteristic peaks accompanied with the satellite peaks (Fig. 3e, Supplementary Fig. 5f), which are attributed to the coexistence of Fe(II) (2*p*_3/2_ at 710 eV and 2*p*_1/2_ at 721 eV) and Fe(III) (2*p*_3/2_ at 714 eV and 2*p*_1/2_ at 724 eV)^34^. The Ni 2p spectrum can be respectively fitted into a pair of peaks with Ni 2*p*_3/2_ at 854 eV and Ni 2*p*_1/2_ at 872 eV (Fig. 3f), which can be assigned to Ni(II)^35^. After the activation, the deconvoluted O 1s spectrum of Ni(Fe)OOH–FeS_*x*_ shows the obviously increased area ratio of M-O to M-OH (Fig. 3c), and the emerged Ni 2*p*_3/2_ (856 eV) and Ni 2*p*_1/2_ (874 eV) peaks are related to NiOOH (Fig. 3f)^36,37^. Moreover, the peak assigned to S–O in the S 2*p* spectrum of Ni(Fe)(OOH)–FeS_*x*_ is shifted and weakened. All the above results suggest the oxidative transformation of Ni(Fe)(OH)_2_–FeS_*x*_ to Ni(Fe)OOH–FeS_*x*_, which is verified by the structural characteristics (Figs. 2 and 3)^38^.

### X-ray absorption fine structure analysis of corrosion products

**Fig. 4:**
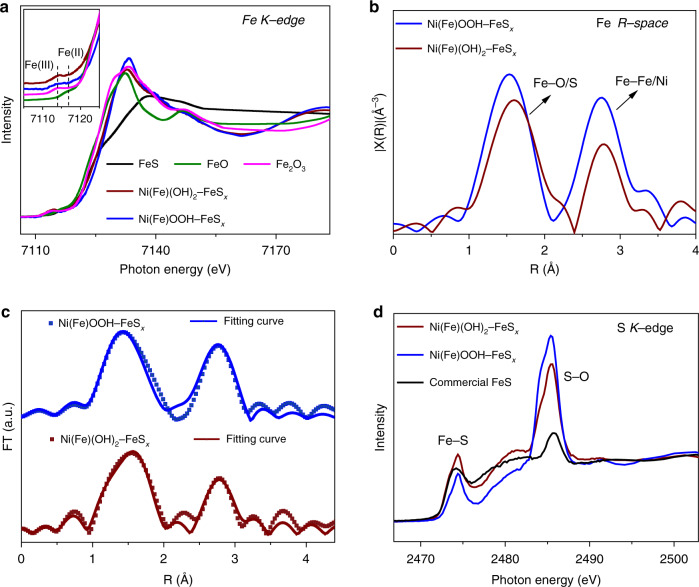
XANES and EXAFS analysis of Ni(Fe)(OH)_2_–FeS_*x*_ and Ni(Fe)OOH–FeS_*x*_. **a** Fe *K*-edge XANES spectra (inset shows the enlarged edge area), **b** Fe *R*-space EXAFS spectra, **c** the associated Fourier transforms and fitting curves of Fe *K*-edge EXAFS spectra, and **d** S *K*-edge XANES spectra.

The structural properties of Ni(Fe)(OH)_2_–FeS_*x*_ and Ni(Fe)OOH–FeS_*x*_ are further studied by X-ray absorption fine structure (XAFS). The Fe *K*-edge X-ray absorption near edge structure (XANES) shows that the Fe(II) and Fe(III) species coexist in the Ni(Fe)(OH)_2_–FeS_*x*_ and Ni(Fe)OOH–FeS_*x*_. Notably, the intensity of main absorption peak of Ni(Fe)OOH–FeS_*x*_ exceeds that of Ni(Fe)(OH)_2_-FeS_*x*_, and the energy of the rising edge is higher in the former, which suggests that a higher oxidation state of Fe in the activated Ni(Fe)OOH–FeS_*x*_ (Fig. 4a). The Fe–Fe/Ni and Fe–O/S bonds of Ni(Fe)(OH)_2_–FeS_*x*_ and Ni(Fe)OOH–FeS_*x*_ are verified by the Fe *R*-space extended X-ray absorption fine structure (EXAFS) profiles (Fig. 4b). Compared with the larger coordination number (CN) of Fe–O, Fe–S and Fe-Ni in the Ni(Fe)OOH–FeS_x_ (CN_Fe–O_ = 2.9, CN_Fe–S_ = 2.6, CN_Fe–Ni_ = 4.7), Ni(Fe)(OH)_2_–FeS_*x*_ shows smaller CN_Fe–O_ (1.7), CN_Fe–S_ (1.0) and CN_Fe–Ni_ (1.8) (Fig. 4c, Supplementary Table 2), indicating the defective local atomic structural and distortion in the activated Ni(Fe)OOH–FeS_*x*_^39^. The defective/distorted structure revealed by EXAFS is in accordance with the charge state determined by XANES analysis (Fig. 4a). Moreover, the presence of Fe–S bonds in the Ni(Fe)(OH)_2_–FeS_*x*_ and Ni(Fe)OOH–FeS_*x*_ is further confirmed by the S *K*-edge XANES spectra (Fig. 4d). Compared with the initial Ni(Fe)(OH)_2_–FeS_*x*_, the decreased content of Fe–S and the enhanced content of S-O in the Ni(Fe)OOH–FeS_*x*_ suggest some partially oxidized S species. Nevertheless, most Fe–S species are preserved in the Ni(Fe)OOH–FeS_*x*_.

### Electrochemical performance of corrosion electrodes

**Fig. 5:**
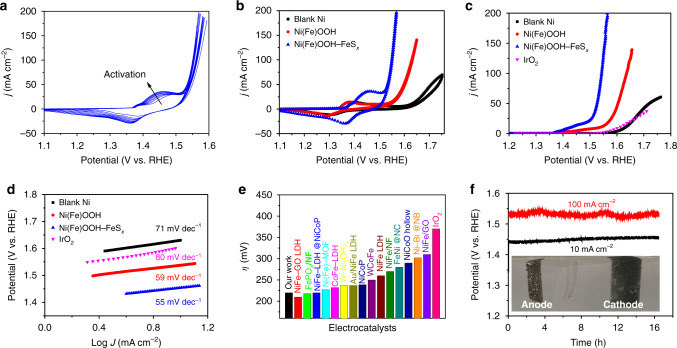
Electrochemical measurements of different electrodes. **a** Activation CV curves of Ni(Fe)OOH–FeS_*x*_. **b** CV profiles, **c** polarization curves, and **d** the corresponding Tafel plots of different electrodes, **e** activity comparison of recent NiFe-based electrocatalysts. **f** Chronopotentiometric curves of Ni(Fe)OOH–FeS_*x*_ obtained at the constant current densities of 10 and 100 mA cm^–2^ (inset shows the three electrodes photograph).

The activation process is completed after only 10 cycles of cyclic voltammetry (CV) scans, which is associated with the phase transform from hydroxides to oxyhydroxides (Fig. 5a). The peak pair in the potential range of 1.25–1.50 V versus the reversible hydrogen electrode (vs. RHE) corresponds to the redox couple of Ni(II)/Ni(III)^40^. Fig. 5b shows the CV curves of three electrodes after the activation process. For comparison, Fig. 5c shows the linear scan polarization curves for oxygen evolution over different electrodes. Among them, the blank Ni foam exhibits a similar activity (an overpotential (*η*) of 400 mV at 10 mA cm^−2^) to the commercial IrO_2_ catalyst (an *η* of 370 mV at 10 mA cm^−2^). After the corrosion treatment, the resultant Ni(Fe)OOH electrode demonstrates a significant enhancement in activity as it requires an *η* of only 300 mV to reach the current density of 10 mA cm^−2^. Remarkably, the microorganism-assisted corrosion-formed Ni(Fe)OOH–FeS_*x*_ electrode exhibits a greatly enhanced activity, requiring an *η* of only 220 mV to achieve the current density of 10 mA cm^-2^. As seen in the corresponding Tafel plots (Fig. 5d), the Ni(Fe)OOH and Ni(Fe)OOH–FeS_*x*_ electrodes respectively exhibit the Tafel slopes of 59 and 55 mV dec^−1^, which is much lower than that of the blank Ni foam electrode (71 mV dec^−1^) and IrO_2_ (80 mV dec^−1^). Apparently, the Ni(Fe)OOH–FeS_*x*_ electrode shows the most favorable kinetics for oxygen evolution. Compared to recent NiFe-based materials, this Ni(Fe)OOH–FeS_*x*_ electrode shows a comparable performance for the oxygen evolution (Fig. 5e, Supplementary Table 3). The electrochemically active surface area (ECSA) is measured by the double-layer capacitance via CV curves in the double-layer region at different scan rates (Supplementary Fig. 6). Both calculated capacitances of Ni(Fe)OOH–FeS_*x*_ (3.1 mF cm^−2^) and Ni(Fe)OOH (2.3 mF cm^−2^) are higher than that of the blank Ni electrode (1.9 mF cm^−2^). This suggests that the increased active sites and enhanced intrinsic activity of NiFe-based nanosheets play the dominant role in improving the activity. Electrochemical impedance spectroscopy (EIS) at 1.54 V is applied to further obtain the charge-transfer kinetics of the electrocatalytic process. The charge-transfer resistances (*R*_*ct*_) of the Ni(Fe)OOH–FeS_*x*_ (2.2 Ω) and Ni(Fe)OOH (10 Ω) electrodes are much lower than that of the blank Ni foam electrode (35 Ω), suggesting the favorable charge transfer capability over these corrosion-formed electrodes (Supplementary Fig. 7a). Moreover, the Ni(Fe)OOH–FeS_*x*_ electrode displays a high Faradaic efficiency of 97% for oxygen production (Supplementary Fig. 7b). The Ni(Fe)OOH–FeS_*x*_ electrode can function steadily in 1.0 M KOH electrolyte for at least 16 h at constant current densities of 10 mA cm^−2^ at ~1.45 V and 100 mA cm^−2^ at ~1.53 V (Fig. 5f, Supplementary Movies 1–4). Furthermore, no significant structural changes are observed on the corrosion-formed electrode after the stability test (Supplementary Fig. 8).

The effects of the SRB amount (*V*_SRB_, 23%, 33% and 65%) and corrosion time (3–17 days) are investigated to monitor the corrosive environment (Supplementary Fig. 9). In general, the electrodes produced by simple chemical corrosion show enhanced performance, and the electrodes obtained by SRB-assisted corrosion show further enhanced performance (Supplementary Fig. 9f). For different SRB contents, a volume fraction (*V*_*SRB*_) of 33% appears most favorable to produce highly active electrodes. With different corrosion time, the electrocatalytic activity of electrodes first increases and then decreases. When the corrosion time is 10 days, the electrocatalytic activity reaches the optimum. FESEM images also verified the surface evolution over the corrosion process (Supplementary Fig. 10). Particularly, the amount of SRB on the corrosion-formed biofilm increases initially with the corrosion time from 3 days to 10 days. After that, the SRB gradually fall off the electrode surface, and the corrosion-formed biofilm becomes looser even cracked (Supplementary Fig. 11), which is generally consistent with the growth trend of SRB^28^. This microorganism-assisted strategy can be effectively extended to various metal substrates (Supplementary Fig. 12) and transition metal compounds (Supplementary Fig. 13).

### Theoretical calculations into different electrodes

**Fig. 6:**
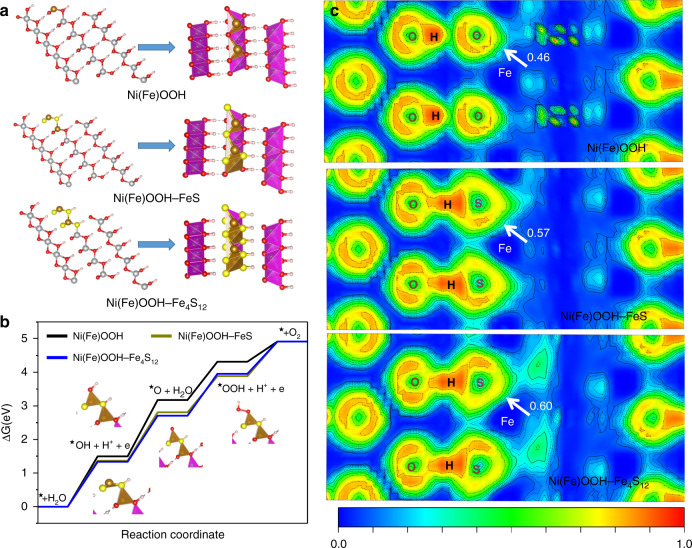
DFT calculations. **a** Structural model, **b** free energy diagram, **c** electron localization function.

To provide further insights into the underlying mechanism for these highly efficient Ni(Fe)OOH–FeS_*x*_ electrodes, some density functional theory (DFT) calculations are performed. The surface substitution of S into transition-metal oxyhydroxides will enhance the electrocatalytic activity, thus the computations of Fe on the surface affected by the surrounding incorporation of S are considered^41,42^. And the structures of Ni(Fe)(OH)_2_–FeS_*x*_ are constructed using the model developed by Friebel and coworkers^43^. Specifically, the O atoms at the surface and subsurface of Ni(Fe)(OH)_2_ are replaced by the S atoms to form the Fe–S species in Ni(Fe)(OH)_2_–FeS_*x*_, and Ni(Fe)(OH)_2_–Fe_4_S_12_ is optimally built by replacing the Fe_4_O_12_ with Fe_4_S_12_ cluster (Supplementary Figs. 14–16). After the activation, the protons and S species of Ni(Fe)(OH)_2_–FeS_*x*_ are respectively subtracted and partially oxidized to form Ni(Fe)OOH–FeS_*x*_ (Fig. 6a). A well-established four-step mechanism is employed to investigate the reaction kinetics. Thermodynamic analyses suggest that the formation of *O intermediate is the rate-determining step (RDS) on these corrosion electrodes (Fig. 6b, Supplementary Fig. 17a and Table 4). The surface oxidation simultaneously occurs on the Ni(Fe)(OH)_2_ during the electrochemical activation process, and the lower free energy gap between *O and *OOH will lead to a reduced *η* of Ni(Fe)OOH than that of the corresponding Ni(Fe)(OH)_2_, which means the oxygen evolution catalyzed by Ni(Fe)OOH will be more favorable than Ni(Fe)(OH)_2_ (Fig. 6b, Supplementary Table 4). Moreover, the incorporation of S into Ni(Fe)(OH)_2_-FeS_x_ and Ni(Fe)OOH–FeS_*x*_ will induce a further decreased free energy gap between *O and *OOH and lead to a reduced *η* for the oxygen evolution (Supplementary Fig. 17a, b). As the increased S substitution on the surface of Ni(Fe)OOH, the decreased free energy gap between *OH and *O will lead to a further reduced *η* on the activated Ni(Fe)OOH–FeS_*x*_ (Fig. 6b, Supplementary Table 4). These results suggest hydroxyl sulfide or sulfurized hydroxide would promote the oxygen evolution on the Ni(Fe)OOH–FeS_x_. By analyzing the density of state (DOS), it is found that the *η* is correlated with the net charge of Fe (Supplementary Fig. 18, Table 5). Compared with the net charge of Fe (0.227) and Fermi level (−2.46) of Ni(Fe)(OH)_2_, the larger net charge of Fe (0.287) and lower Fermi level (−2.67) of Ni(Fe)(OH)_2_–Fe_4_S_12_ suggests a higher adsorption capability to the reaction intermediate. After the electrochemical activation, the net charge of Fe (0.496) and Fermi level (−2.88) of Ni(Fe)OOH–Fe_4_S_12_ is significantly improved as the surface oxidation. Compared with the electron localization function (ELF) of Fe–O bonds (0.43) for Ni(Fe)(OH)_2_, the incorporation of S will induce a higher ELF of 0.51 for the Fe–S bonds in Ni(Fe)(OH)_2_–Fe_4_S_12_ (Supplementary Fig. 17c, d), which indicates Fe–S bonds are more ionic than Fe–O bonds. After the electrochemical activation, the ELF of Fe–S bonds in Ni(Fe)OOH–Fe_4_S_12_ is further increased to 0.60. With the increased S substitution on the surface of Ni(Fe)OOH, the gradual electron localization will lead to a further increased ELF of Fe–S bonds in the activated Ni(Fe)OOH–FeS_*x*_ (Fig. 6c), resulting in more negative charge on the S atoms and more positive charge on the Fe atoms. Moreover, a stronger hydrogen bond interaction of O-H..S than that of O-H:::O would lead to more charge transfer from S to H atoms and more positive charge existed on the Fe atoms. Therefore, it can improve the bonding between Fe and *O intermediate and lead to an enhanced oxygen evolution performance^37^.

## Discussion

In summary, this work presents a microorganism corrosion strategy for preparing highly efficient electrodes towards electrocatalytic water oxidation. The corrosion-formed Ni(Fe)OOH–FeS_*x*_ electrode manifests excellent activity for oxygen evolution in alkaline electrolyte, requiring an overpotential of only 220 mV to obtain the benchmark current density of 10 mA cm^−2^. Experimental results suggest that the synergistic effect between Ni(Fe)OOH and Fe–S species would be responsible for this enhanced activity. Theoretical calculations reveal that the rate-determining step of oxygen evolution is the formation of *O on the Ni(Fe)OOH–FeS_*x*_ electrode, the incorporation of Fe–S species would enhance the bonding between Fe and active *O intermediate and significantly reduce the adsorption free energy gap. This work not only provides an efficient electrode for electrocatalytic oxygen evolution but also perhaps more importantly demonstrates an interesting and facile strategy by microorganism-assisted corrosion engineering. This work is a good demonstration that bridges the gap between traditional corrosion engineering and emerging electrochemical energy conversion technologies. Therefore, the present work is likely to stimulate high interest in the multidisciplinary integration among biology, industrial corrosion, nanomaterials design, and modern energy technologies.

## Methods

### Microbe cultivation and inoculation

In this study, SRB was isolated from the sludge in Sinopec oilfield, China. Compared with sequences in the GenBank database with BLAST program, SRB belongs to Desulfotomaculum nigrificans (*D. nigrificans*). As a result, SRB seed culture was cultivated separately in different media at 37 °C (Supplementary Table 1). First, the culture medium was treated and sterilized at 121 °C for 20 min; Second, vials were inoculated with 100 mL of seed culture and 1000 mL of sterile culture, and then incubated at 37 °C. SRB seed culture was obtained after the incubation for 3 day. The initial concentration of SRB inoculum was 8.0 × 10^4^. The amount of SRB (*V*_microbe_) was described as: $V_{{\mathrm{microbe}}} = \frac{{V_{{\mathrm{inoculated}}\;{\mathrm{microbe}}}}}{{V_{{\mathrm{inoculated}}\;{\mathrm{microbe}}} + V_{{\mathrm{sterile}}\;{\mathrm{culture}}}}} \times {\mathrm{100\% }}$

### Corrosion electrode preparation

Ni foam (1.0 × 1.0 cm^2^) and Ti sheet (1.0 × 1.0 cm^2^) were pretreated in acetone and ethanol (volume ratio is 1:1) for 30 min to remove the impurities attached on the electrode surface, then put them into 3.0 M HCl solution for 15 min to remove the oxide layers on their surface, and rinsed subsequently with water and ethanol, finally dried with nitrogen gas before use. The corrosion electrode was prepared by putting the pretreated nickel foam into a reagent bottle containing different *V*_SRB_ (23%, 33% and 65%) of inoculated SRB, then kept them in an anaerobic environment and incubated at 37 °C. Considering the growth curve of SRB, the corrosion time was set to 3 days, 7 days, 10 days, 14 days and 17 days. For comparison, blank Ni foam was treated with the sterile medium at 37 °C without adding SRB in the corrosion procedure. Furthermore, to investigate the characteristics of pure biofilm, the blank Ni foam was replaced by non-corroding Ti sheets in the same corrosive environment with SRB. After taking out from the SRB corrosion system, the electrodes were rinsed by distilled water and cleaned by absolute ethanol to remove the residual SRB, and dried under a nitrogen gas stream. Similarly, various commercial metal substrates (Fe foam, Cu foam, Co sheet, NiFe alloy foam, NiCo alloy foam, stainless, carbon steel and NiFeCu) as well as reported transition metal compound electrodes (CoP/Cu foam, CoP/Fe foam, FeP/Fe foam, Fe_2_O_3_/Fe foam, NiP/Carbon fiber and NiFe-LDH) were also treated in the SRB corrosion system.

## Supplementary information

Supplementary Information

Peer Review File

Description of Additional Supplementary Files

Supplementary Movie 1

Supplementary Movie 2

Supplementary Movie 3

Supplementary Movie 4

## Data Availability

The data that support the findings of this study are available from the corresponding author upon reasonable request.
