# Supplementary material for: Preparation of nickel-iron hydroxides by microorganism corrosion for efficient oxygen evolution
Source: Nat Commun. 2020 Oct 8;11:5075. doi: 10.1038/s41467-020-18891-x (PMC7545195; doi:10.1038/s41467-020-18891-x)
Supplement: Supplementary file 1 — Supplementary Information [file 41467_2020_18891_MOESM1_ESM.docx]

**Preparation of Nickel-Iron Hydroxides by Microorganism Corrosion for Efficient Oxygen Evolution**

*Huan Yang,^a^ Lanqian Gong,^a^ Hongming Wang,^b^ Chungli Dong, ^c^ Junlei Wang, ^a^ Kai Qi,^a^ Hongfang Liu,^a^ Xingpeng Guo,^a^* and *Bao Yu Xia ^a,^ **

*^a^* Key Laboratory of Material Chemistry for Energy Conversion and Storage (Ministry of Education), Hubei Key Laboratory of Material Chemistry and Service Failure, Wuhan National Laboratory for Optoelectronics, School of Chemistry and Chemical Engineering, Huazhong University of Science and Technology (HUST), 1037 Luoyu Road, Wuhan 430074, China

*^b^* Institute for Advanced Study, Nanchang University, 999 Xuefu Road, Nanchang, China

*^c^* Department of Physics, Tamkang University, 151 Yingzhuan Road, New Taipei City 25137, Taiwan, China

Correspondence email: [byxia@hust.edu.cn](mailto:byxia@hust.edu.cn) (B.Y.Xia)

**Supplementary Methods**

**Physical characterization.** The morphology of various electrodes was observed by field-emission scanning electron microscopy (FESEM, JSM-7600F, Japan) and transmission electron microscopy (TEM, Tecnai G2 F30, Netherlands). The composition of various electrodes was determined using energy-dispersive spectroscopy (EDS, Tecnai G2 F30, Netherlands). X-ray diffraction (XRD, Empyrean, Netherlands) was measured with Cu Ka radiation (λ=1.5416 Å). Raman spectra (HR800, France) were collected at the wavelength of 532 cm^-1^. The synchrotron X-ray spectroscopies were performed at the National Synchrotron Radiation Research Center (NSRRC), Taiwan. The extended x-ray absorption fine structure (EXAFS) and x-ray absorption near edge structure (XANES) at Fe K-edge were recorded with transmission mode at BL17C. And FeS, FeO and Fe_2_O_3_ were used as the standard samples. The base pressure of X-ray Photoelectron Spectroscopy (XPS, VG Multilab 2000) analyses in the experimental chamber was below 10^-9^ mbar, the spectra were measured with Al Ka (1486.6 eV) radiation and the overall energy resolution was 0.45 eV. The binding energies were calibrated relative to the C 1s peak at 284.6 eV.

**Electrochemical measurements.** The electrochemical tests (repeated for at least five times) were performed on an AutoLab 302N potentiostat/galvanostat electrochemical workstation with three-electrode system at room temperature (~ 25 ^o^C). The as-obtained electrode was used as the working electrode. Graphite rod was used as the counter electrode and a Hg/HgO electrode as the reference electrode. The electrolyte was 1.0 M KOH. Potentials displayed in this work were converted to the reversible hydrogen electrode (RHE) scale using $E_{\mathrm{RHE}}=E_{Hg/HgO}+0.0591\times pH+0.097$, and the overpotential *η* = *E*_RHE_ - 1.23. The linear scan polarization curves were measured at a scan rate of 5.0 mV s^-1^, starting from 1.0 V to 1.7 V vs. RHE. The electrochemical impedance spectroscopy (EIS) was measured at 1.54 V vs*.* RHE. The frequency range was 10^5^ Hz-0.01 Hz, and the internal resistance of solution (*R*_S_) of EIS was applied to the *iR*-compensation for LSV tests. CV measurements were performed in the potential range from 1.07 to 1.18 V vs*.* RHE at different scan rates of 20, 50, 100, 150, 200, 250 mV s^-1^, and 20 cycles were recorded. The electrochemically active surface area (ECSA) of different electrodes was calculated from the CV curves. The stability was tested at constant current densities of 10 mA cm^-2^ and 100 mA cm^-2^.

**Computational methods.** All density functional theory (DFT) calculations were performed with the plane-wave code Vienna Ab-initio Simulation Package (VASP). The surface properties were obtained using the GGA-DFT plus Hubbard-U framework (GGA+U). The spin-polarized RPBE functional was used with an effective U-J term fixed at 6.6 eV for Ni and 3.5 eV for Fe.^1^ The core electron interactions were described using the projector augmented-wave (PAW) pseudopotentials.^2^ The plane wave energy cutoff was set to 400 eV. A Monkhorst-Pack k-point grid of 2×2×1 was chosen to sample the reciprocal space for the calculation systems. At least 15 Å vacuum space between adjacent images was used to prevent the interaction between the replicas along the Z-direction. For the surface adsorption calculations, the slabs were exposed by a 2×3 metal sites with six metal-oxygen layers and the bottom two layers fixed. Electron localization function (ELF) was also applied to analyze the distribution of electrons on various surfaces,^3, 4^ which can be used to describe the electronic localization or delocalization for the material and analyze the bonding type, including covalent, ionic or metallic bond. In the color-coding scheme, a high degree of electron localization is shown in red, whereas the regions of low ELF values (Z) are shown in blue.

For the oxygen evolution reaction, the following four-step mechanism was used to analyze the thermodynamics and the overpotential for oxygen evolution.

H_2_O_(l)_ + * $\rightleftharpoons$ *OH + H^+^ + e^-^

ΔG_1_ = ΔG_*OH_ – eU + k_B_Tln[H^+^] (1)

*OH $\rightleftharpoons$ *O + H^+^ + e^-^

ΔG_2_ = ΔG_*O_ - ΔG_*OH_ – eU + k_B_Tln[H^+^] (2)

H_2_O_(l)_ + *O $\rightleftharpoons$ *OOH + H^+^ + e^-^

ΔG_3_ = ΔG_*OOH_ - ΔG_*O_ – eU + k_B_Tln[H^+^] (3)

*OOH $\rightleftharpoons$ * + O_2(g)_ + H^+^ + e^-^

ΔG_4_ = 4.92 - ΔG_*OOH_ - eU + k_B_Tln[H^+^] (4)

where * represents an active site on the catalyst surface, and *OH, *O and *OOH represent chemisorbed OH, O, and OOH, respectively.

G = E_scf_ + G_solv_ + ZPE – T × S (5)

ΔG_*OH_ = G_*OH_ – (G_*_ + G_H2O(l)_ – 0.5 × G_H2(g)_) (6)

ΔG_*O_ = G_*O_ – (G_*_ + G_H2O(l)_ – G_H2(g)_) (7)

ΔG_*OOH_ = G_*OOH_ – (G_*_ + 2G_H2O(l)_ – 1.5 × G_H2(g)_) (8)

*η*_OER_ (V) = max[ΔG_x_]/e – 1.23

**Supplementary Figures**

**
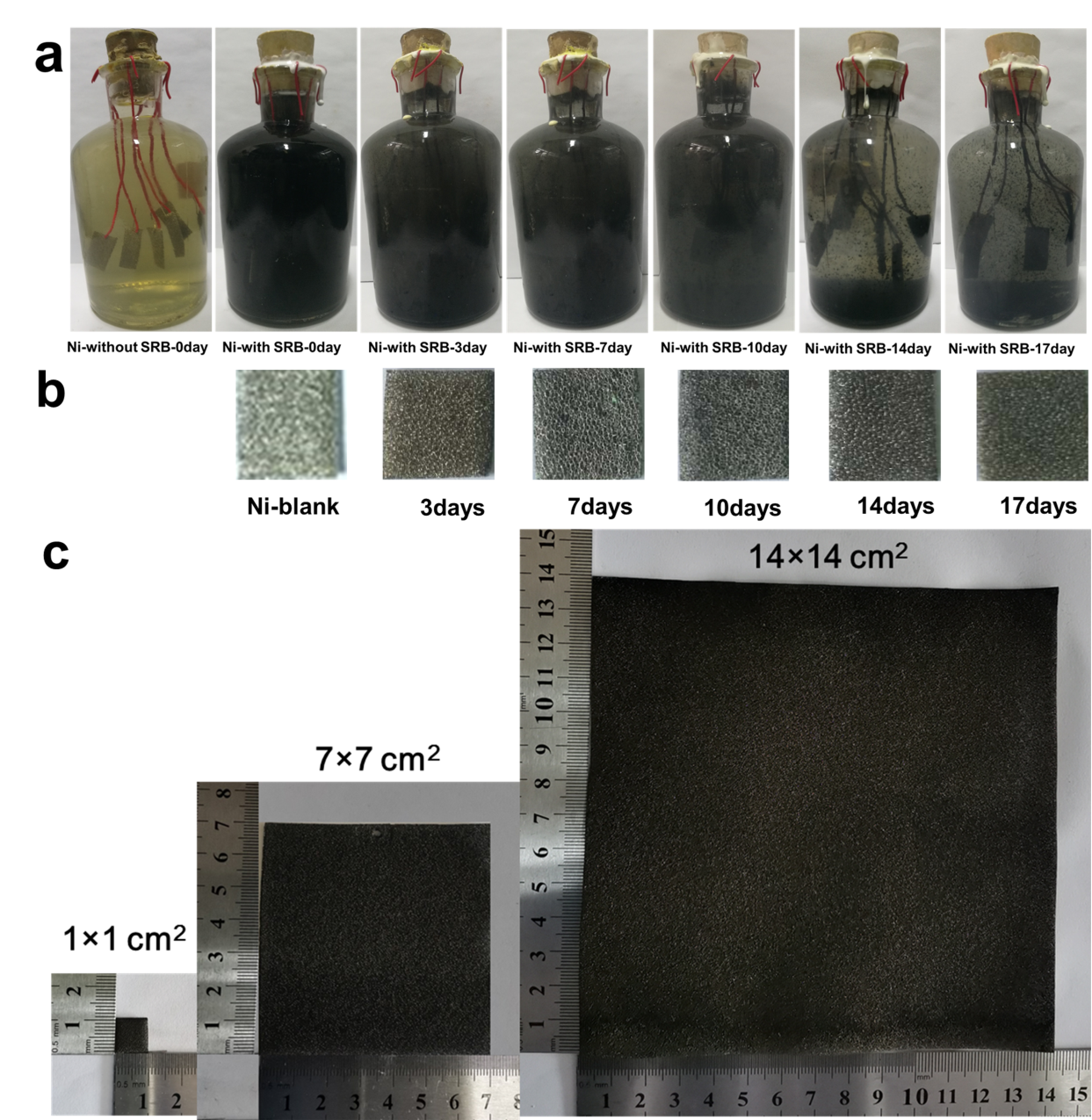
**

**Supplementary Figure 1│Digital images of corrosion electrodes.** Photographs of the corrosion process (a), corrosion electrodes (b) after different times, and (c) corrosion electrodes with different scales (1 × 1 cm^2^, 7 × 7 cm^2^ and 14 × 14 cm^2^).


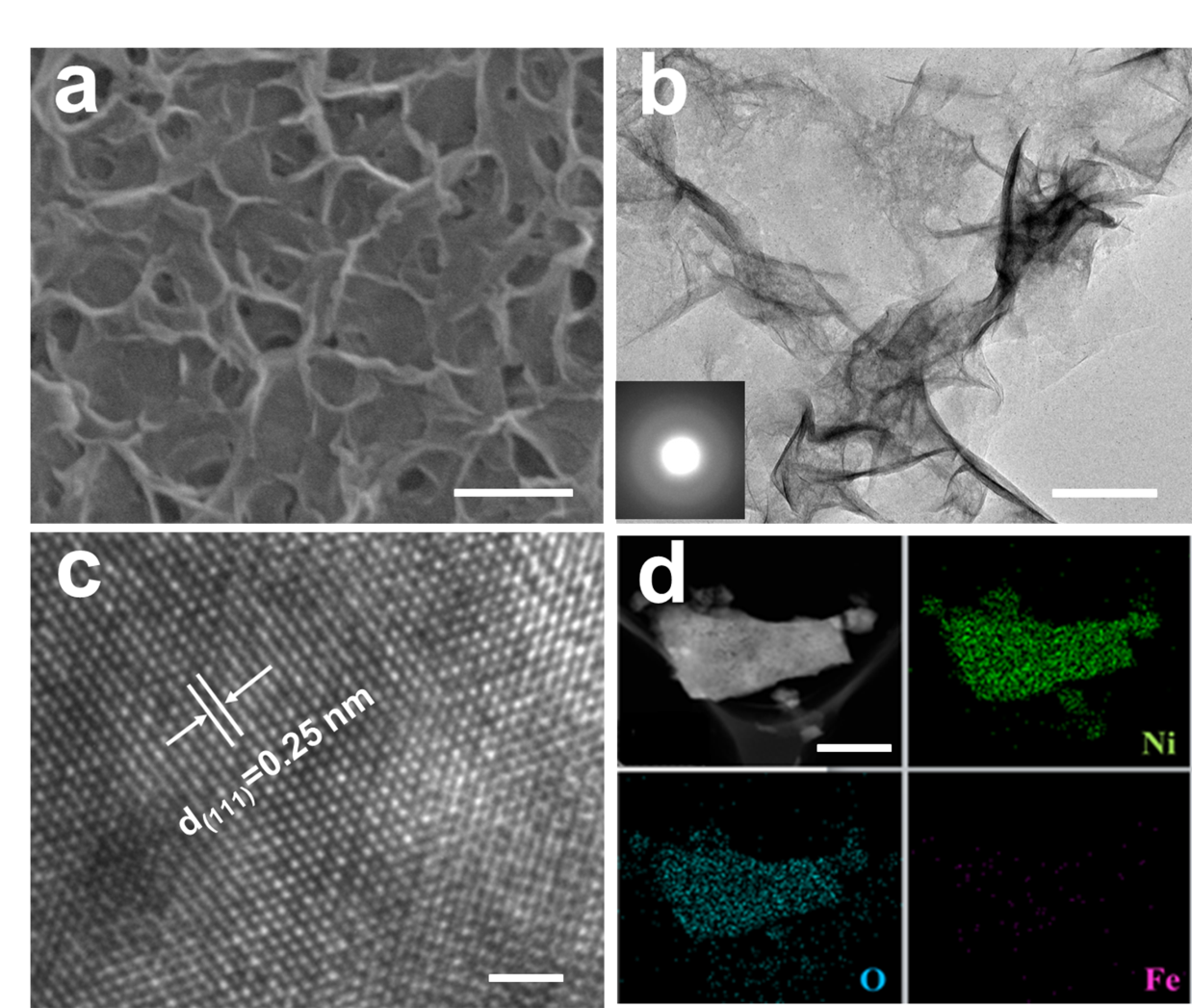


**Supplementary Figure 2│Morphology characterizations of Ni(Fe)(OH)_2_.** (a) FESEM image, scale bar: 200 nm. (b) TEM image and corresponding SAED pattern (inset), scale bar: 100 nm. (c) HRTEM image, scale bar: 2 nm. (d) elemental mappings of Ni(Fe)(OH)_2_ formed in the absence of SRB, scale bar: 500 nm.

**
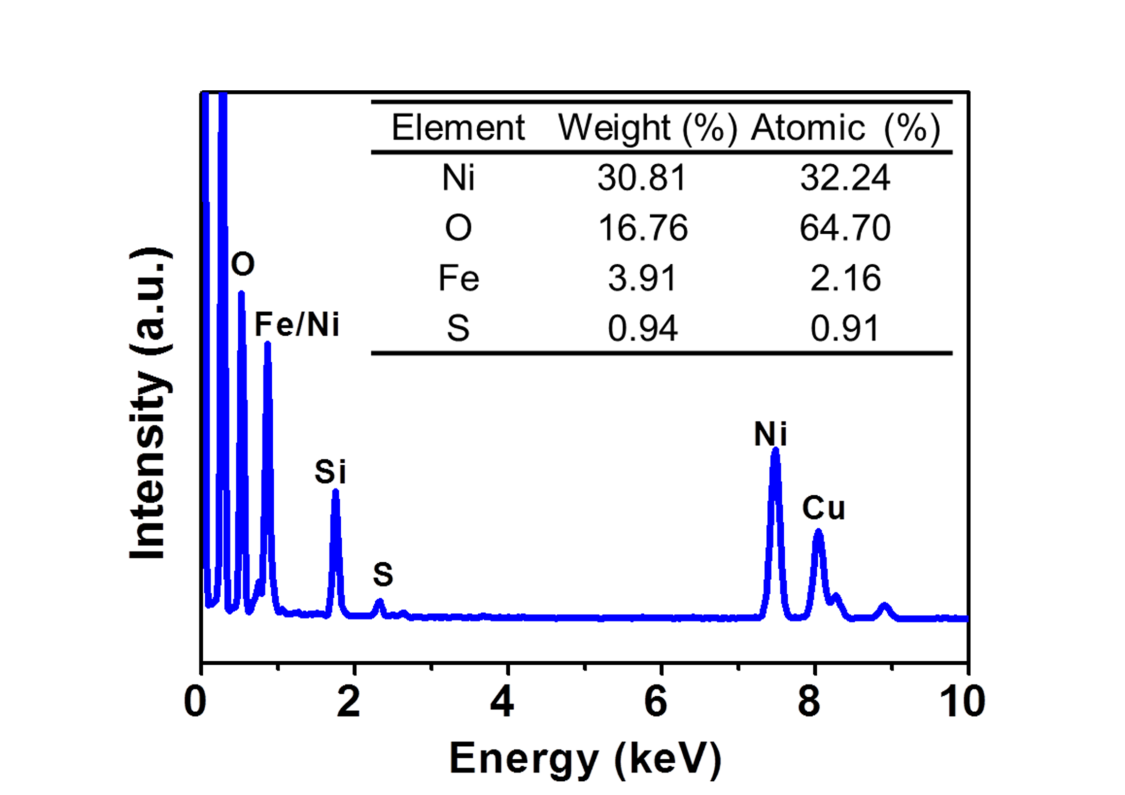
**

**Supplementary Figure 3│EDS analysis of corrosion products.** Corresponding EDS profile of corrosion products (Ni(Fe)OOH-FeS_x_) in Figure 1f. The inset shows the contents of different elements.


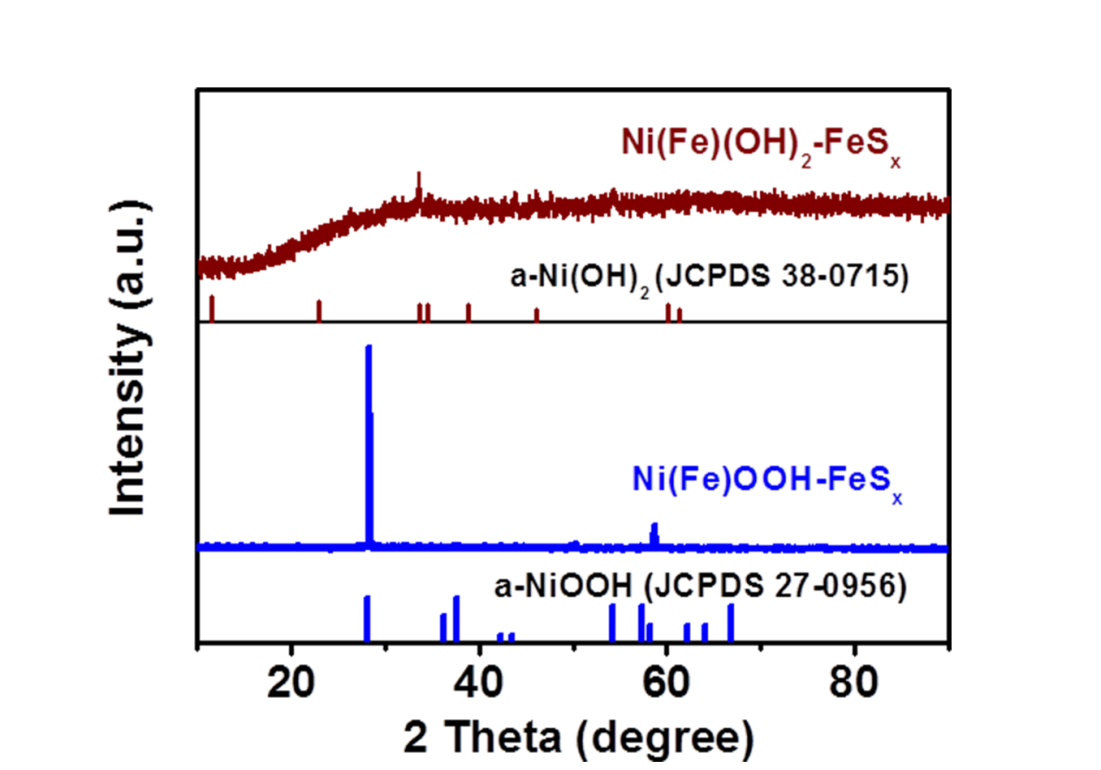


**Supplementary Figure 4│XRD characterizations.** XRD patterns of Ni(Fe)(OH)_2_-FeS_x_ and Ni(Fe)OOH-FeS_x_.


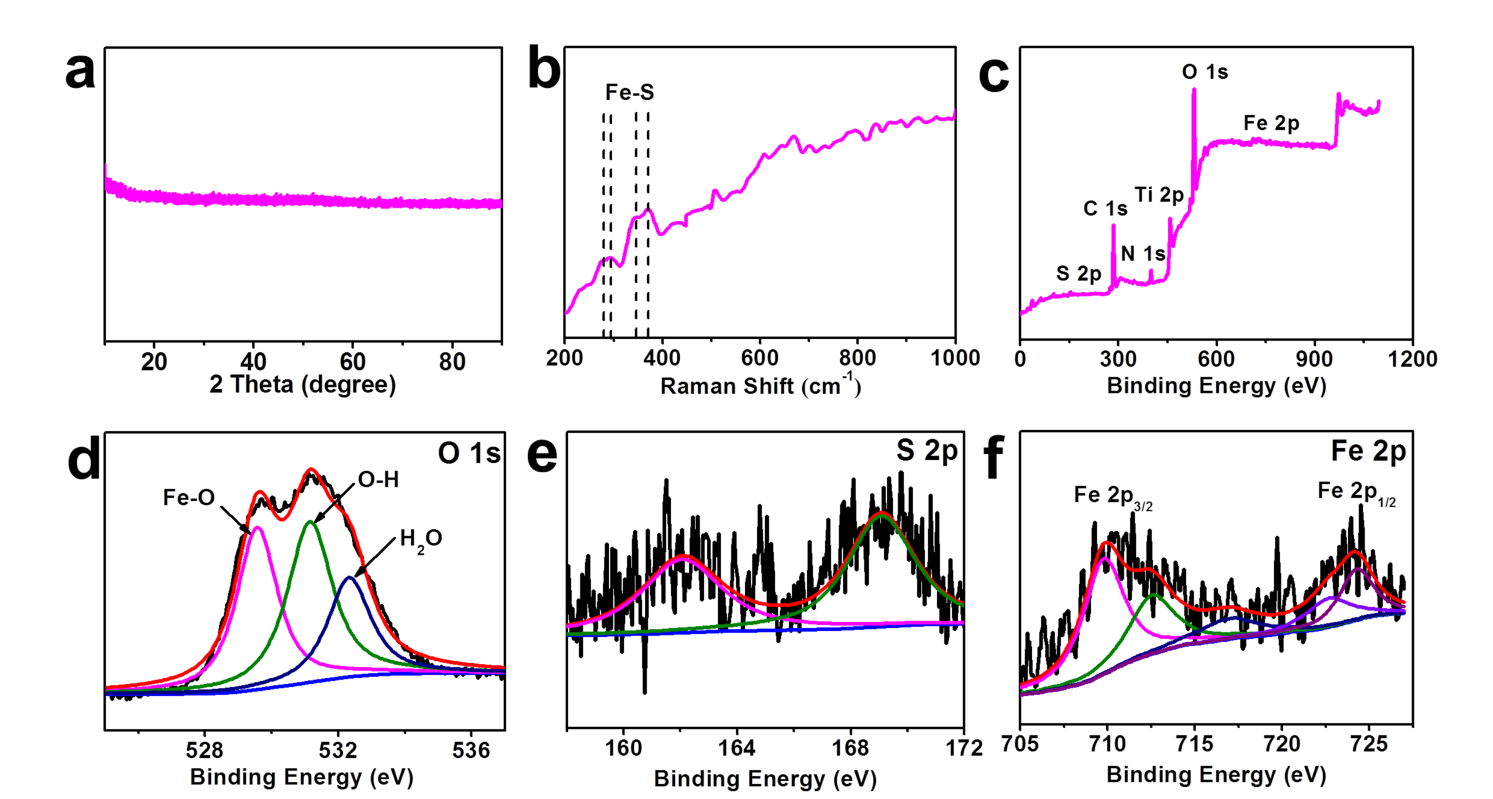


**Supplementary Figure 5│Compositional characterizations.** (a) XRD pattern (subtracted Ti plate), (b) Raman spectrum, (c) XPS survey, high-resolution XPS spectra of (d) O 1s, (e) S 2p, and (f) Fe 2p for treated Ti plate in the SRB corrosion system.


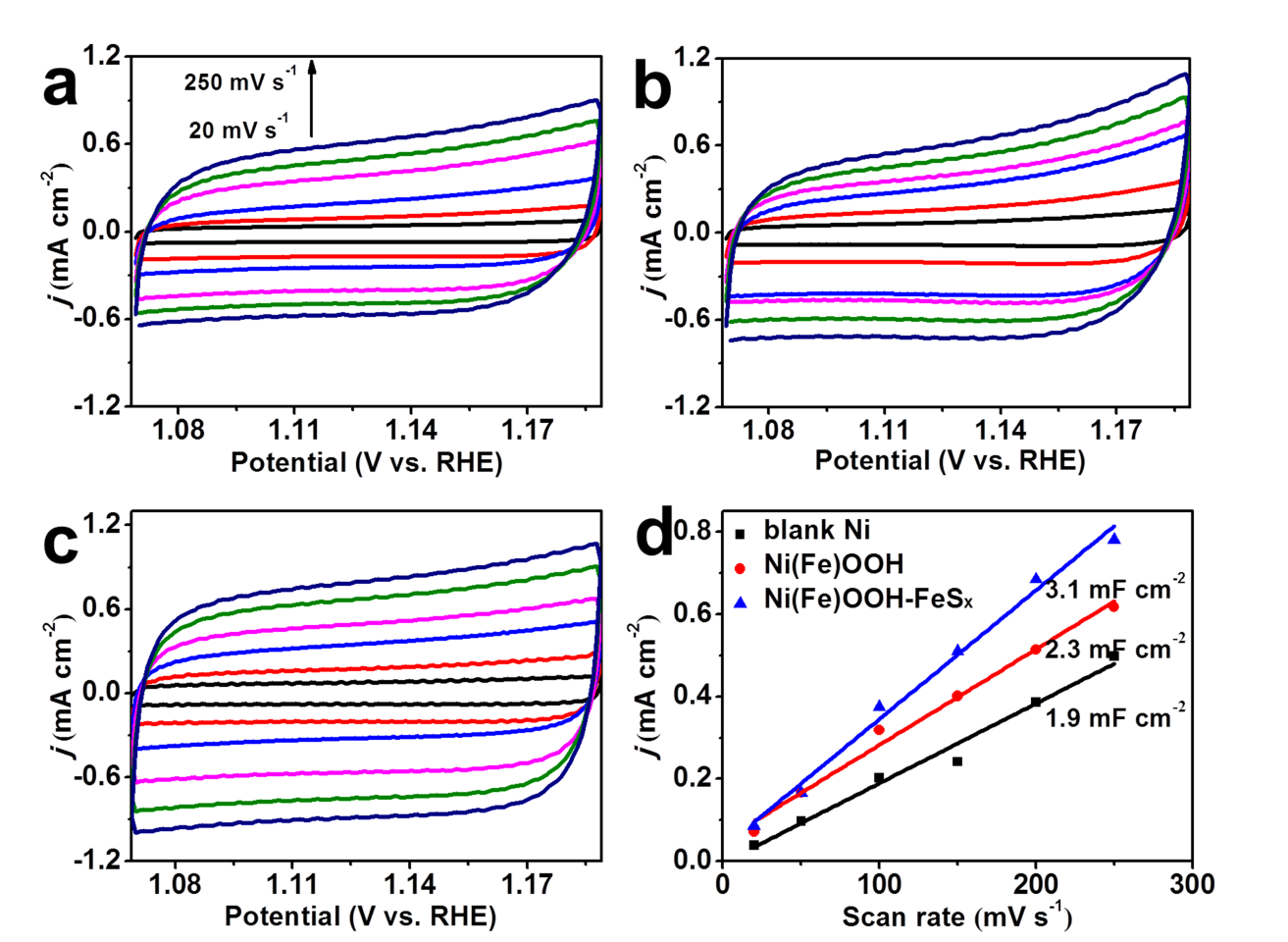


**Supplementary Figure 6│Electrochemical performance of different electrodes.** CV profiles of (a) blank Ni foam, (b) Ni(Fe)OOH, (c) Ni(Fe)OOH-FeS_x_ electrodes, and (d) their capacitive current-scan rate plots.


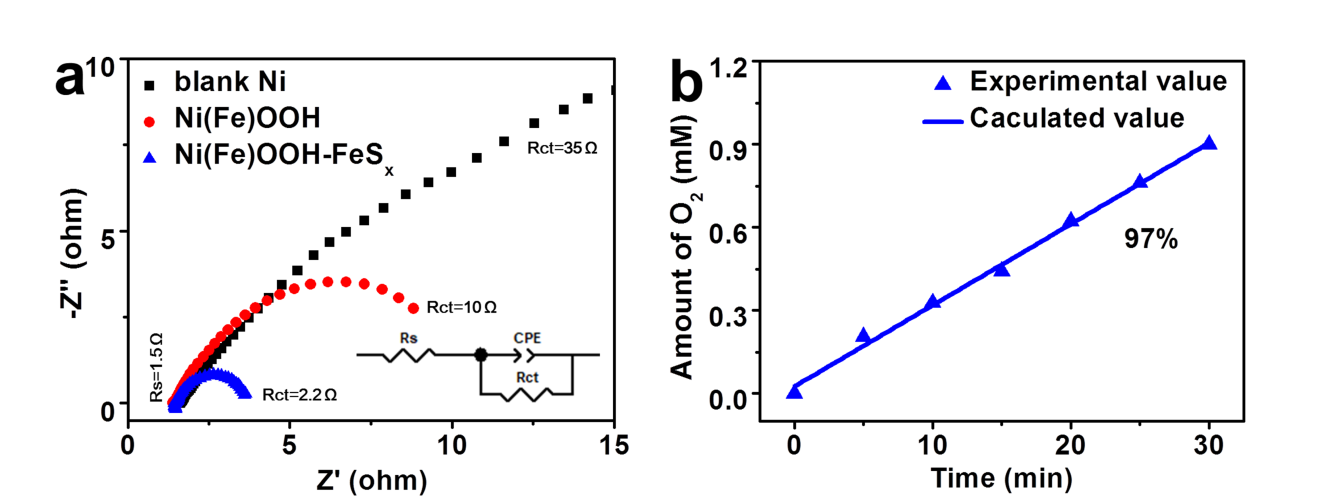


**Supplementary Figure 7│Electrochemical performance of different electrodes.** (a) Nyquist diagrams measured at 1.54 V vs. RHE (inset is the equivalent circuit diagram), and (b) Faradaic efficiency measurements.


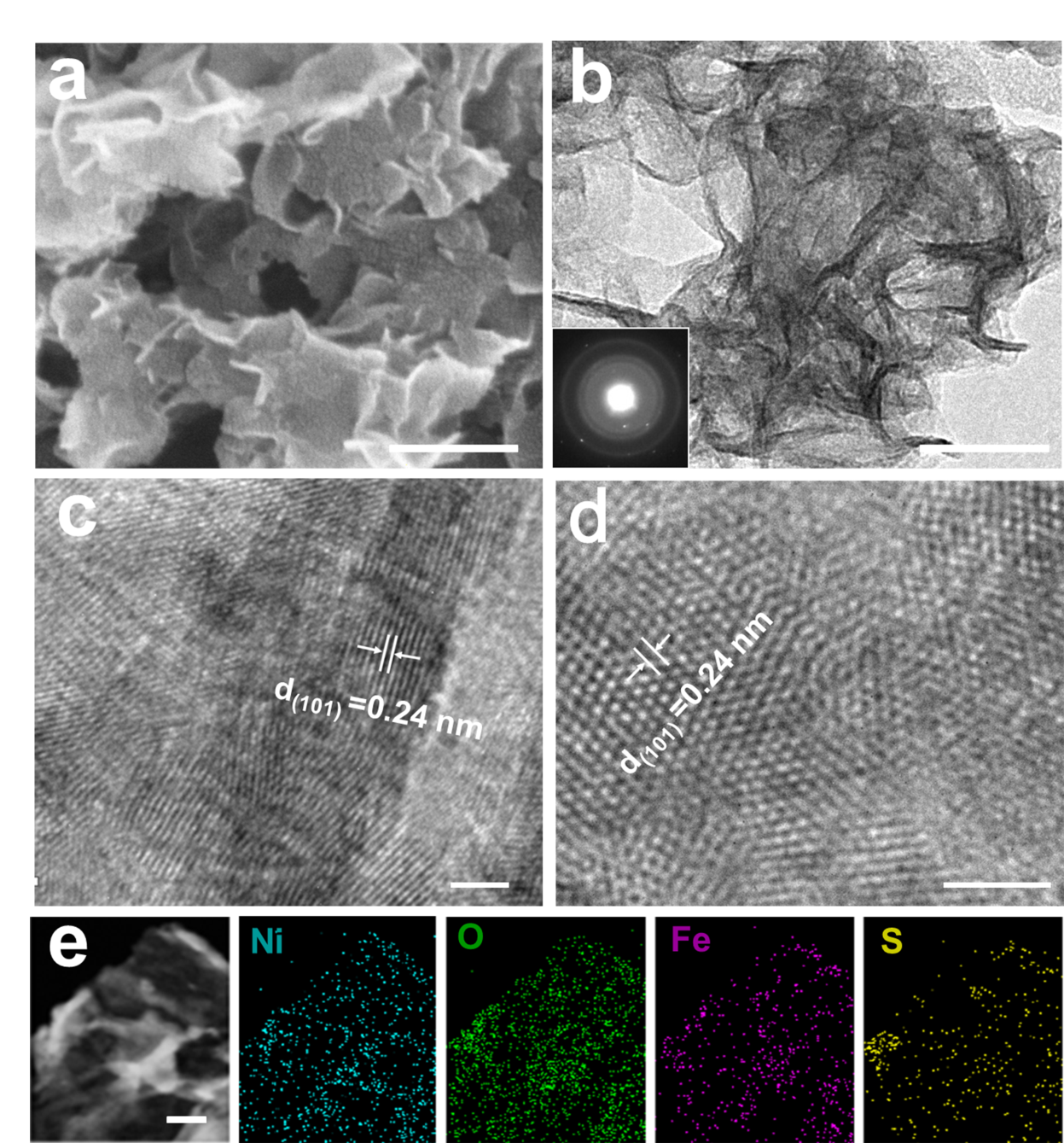


**Supplementary Figure 8│Morphology characterizations.** (a) FESEM image, scale bar: 500 nm. (b) TEM image and corresponding SAED pattern (inset), scale bar: 50 nm. (c, d) HRTEM images, scale bars: 2 nm. (e) elemental mappings of Ni(Fe)OOH-FeS_x_ after the stability test, scale bar: 100 nm.


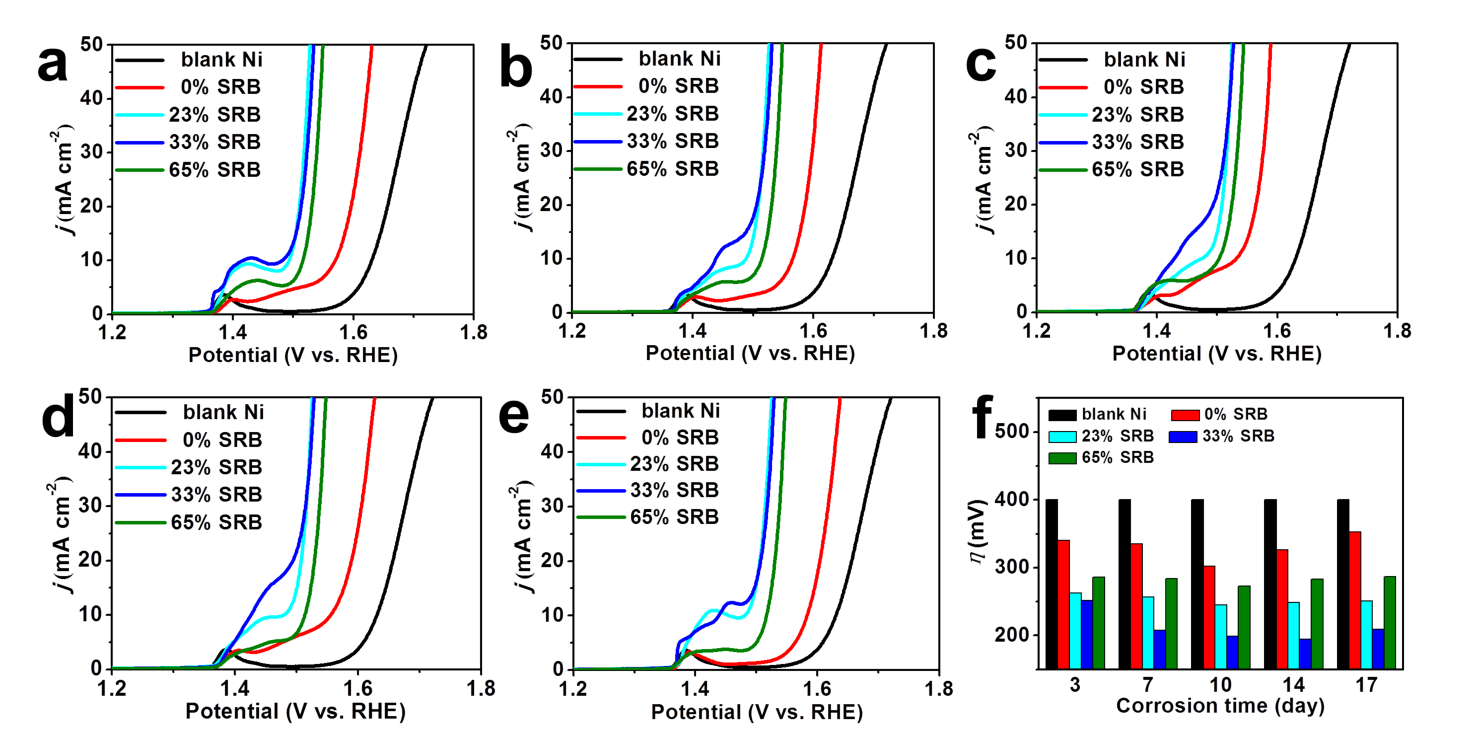


**Supplementary Figure 9│Electrochemical performance of different electrodes.** Polarization curves of Ni foam with different contents of SRB after different corrosion time: (a) 3 days, (b) 7 days, (c) 10 days, (d) 14 days, (e) 17 days, and (f) the corresponding *η* with different contents of SRB at 10 mA cm^-2^.


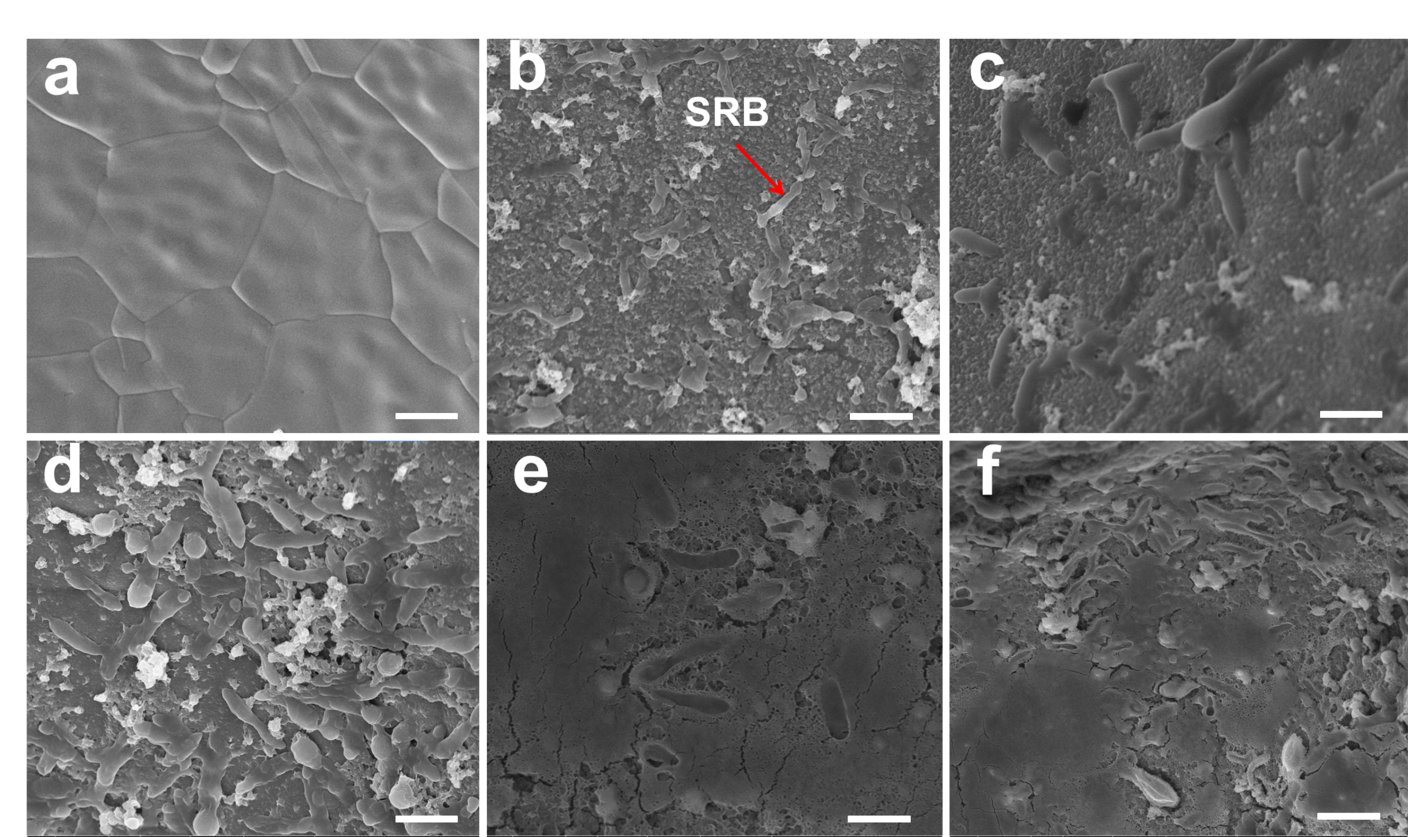


**Supplementary Figure 10│Morphology characterizations.** FESEM images of uncleaned corrosion electrodes after different corrosion time. (a) blank, (b) 3 days, (c) 7 days, (d) 10 days, (e) 14 days, and (f) 17 days. Scale bars: 5 μm (a), 2 μm (b-f).


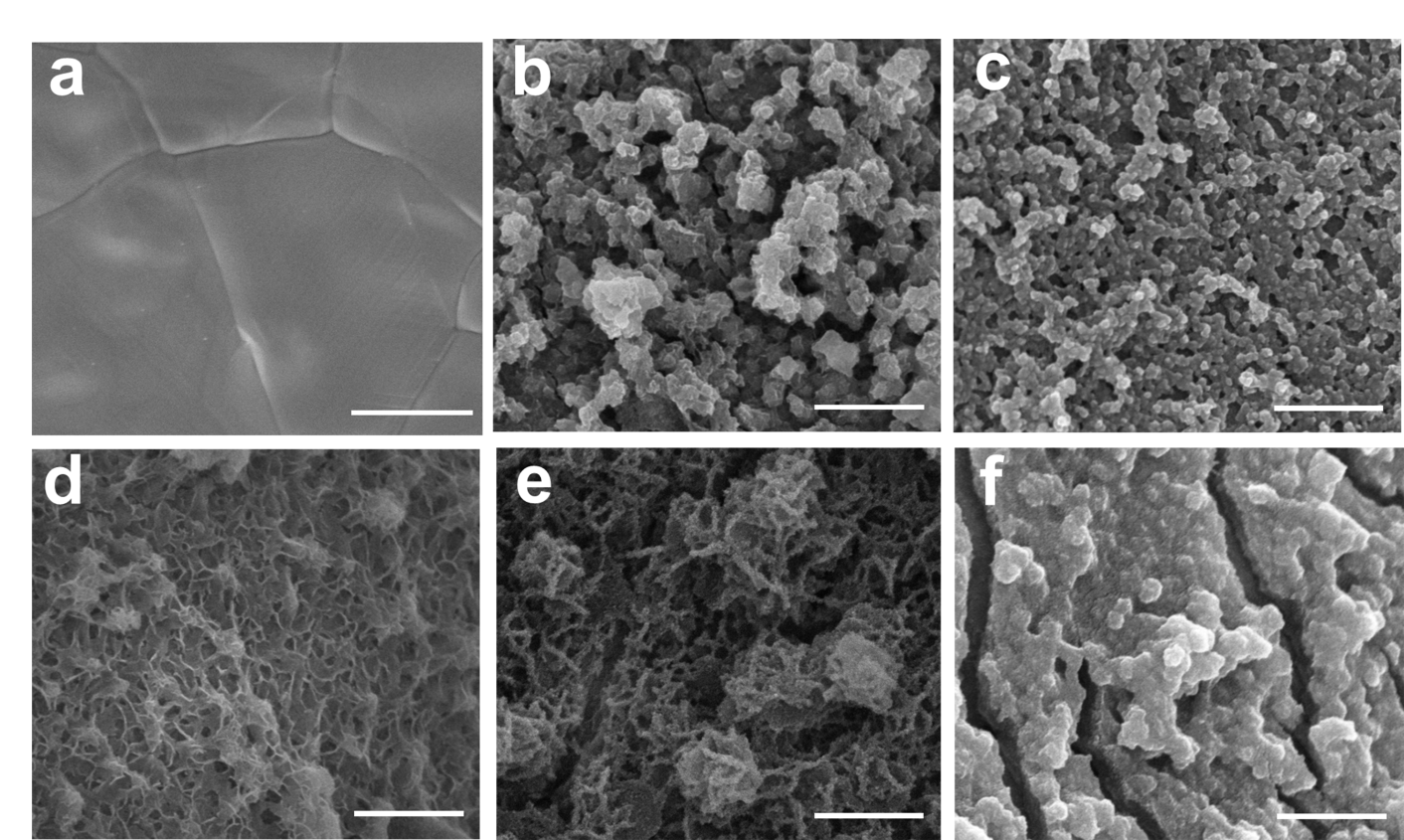


**Supplementary Figure 11│Morphology characterizations.** FESEM images of cleaned corrosion electrodes after different corrosion time. (a) blank, (b) 3 days, (c) 7 days, (d) 10 days, (e) 14 days, and (f) 17 days. Scale bars: 5 μm (a), 500 nm (b-f).

**
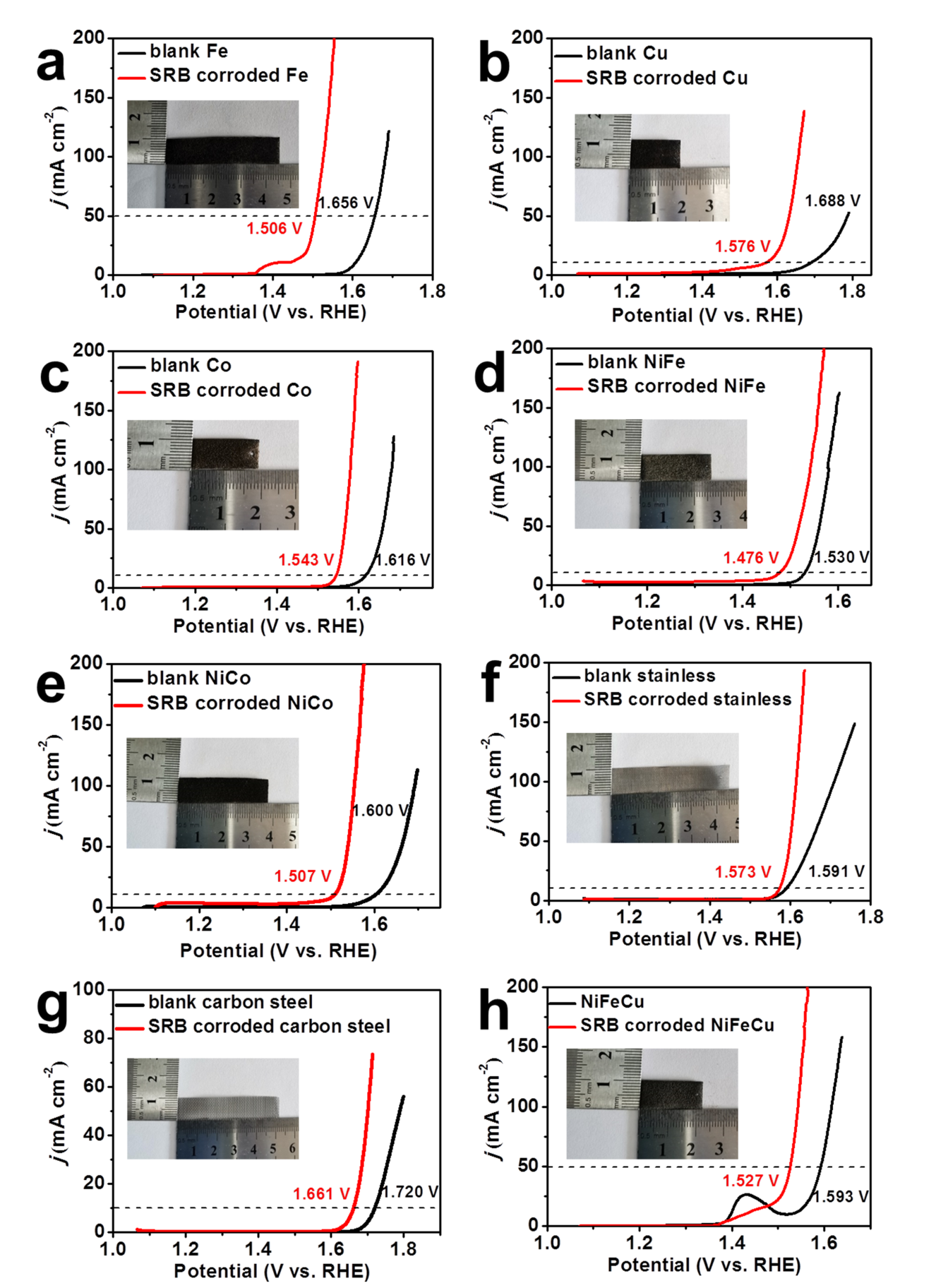
**

**Supplementary Figure 12│Electrochemical performance of different electrodes.** Polarization curves of different commercial metal substrates in the SRB corrosion system and the corresponding photographs of the corrosion electrodes: (a) Fe foam, (b) Cu foam, (c) Co sheet, (d) NiFe alloy foam, (e) NiCo alloy foam, (f) stainless, (g) carbon steel, and (h) NiFeCu (*Nat. Commun.* 2018, 9, 381).


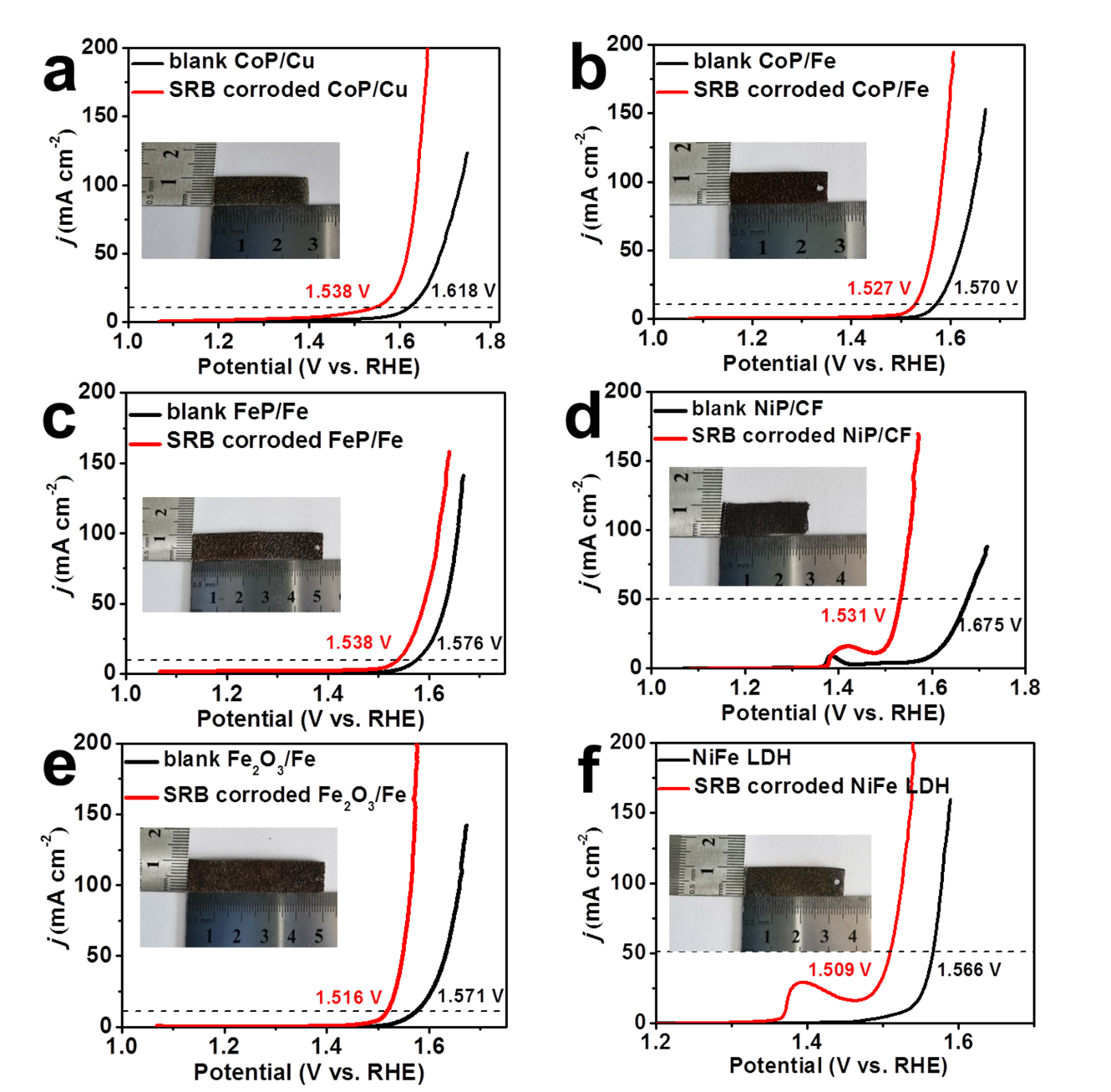


**Supplementary Figure 13│Electrochemical performance of different electrodes.** Polarization curves of the reported OER electrodes treated in SRB corrosion system, inset is the corresponding photographs of corrosion electrodes: (a) CoP/Cu foam, (b) CoP/Fe foam (*J. Mater. Chem. A* 2016, 4, 18272), (c) FeP/Fe foam, (d) Fe_2_O_3_/Fe foam (*Chem. Commun.* 2019, 55, 2513), (e) NiP/Carbon fiber (*J. Mater. Chem. A* 2016, 4, 9691), and (f) NiFe-LDH (*Energy. Environ. Sci.* 2019, 12, 572).


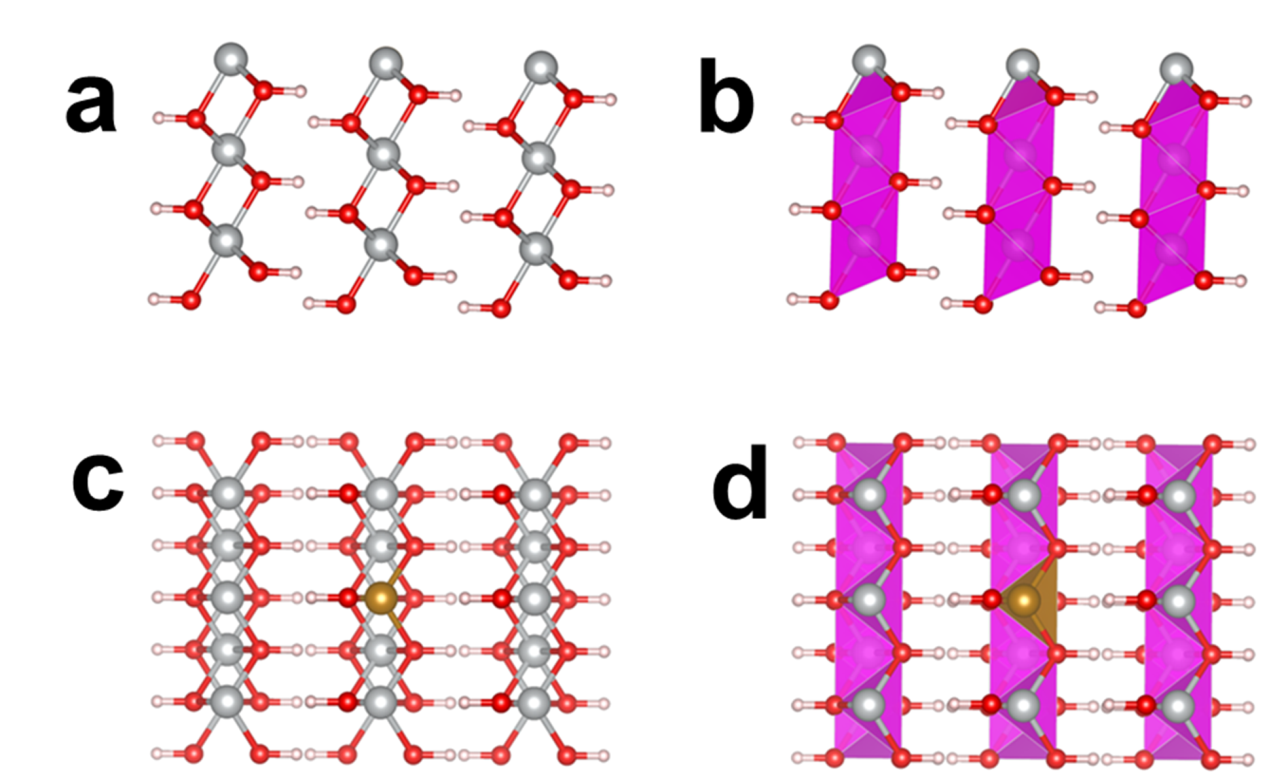


**Supplementary Figure 14│Structure models of Ni(Fe)(OH)_2_.** Side view of ball-and-stick (a) and polyhedral model (b), top view of ball-and-stick (c) and polyhedral model (d).

**
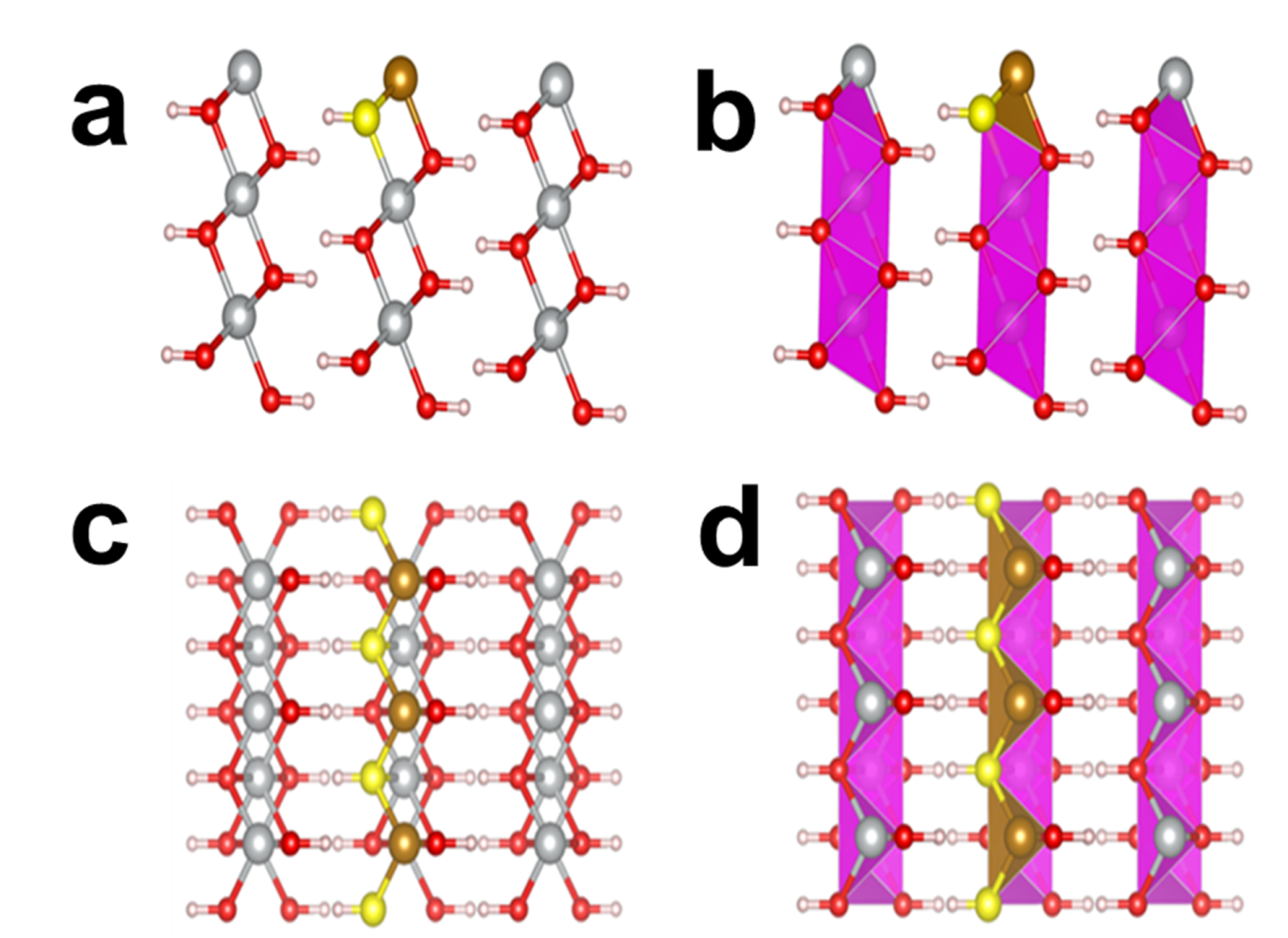
**

**Supplementary Figure 15│Structure models of Ni(Fe)(OH)_2_-FeS.** Side view of ball-and-stick (a) and polyhedral model (b), top view of ball-and-stick (c) and polyhedral model (d).

**
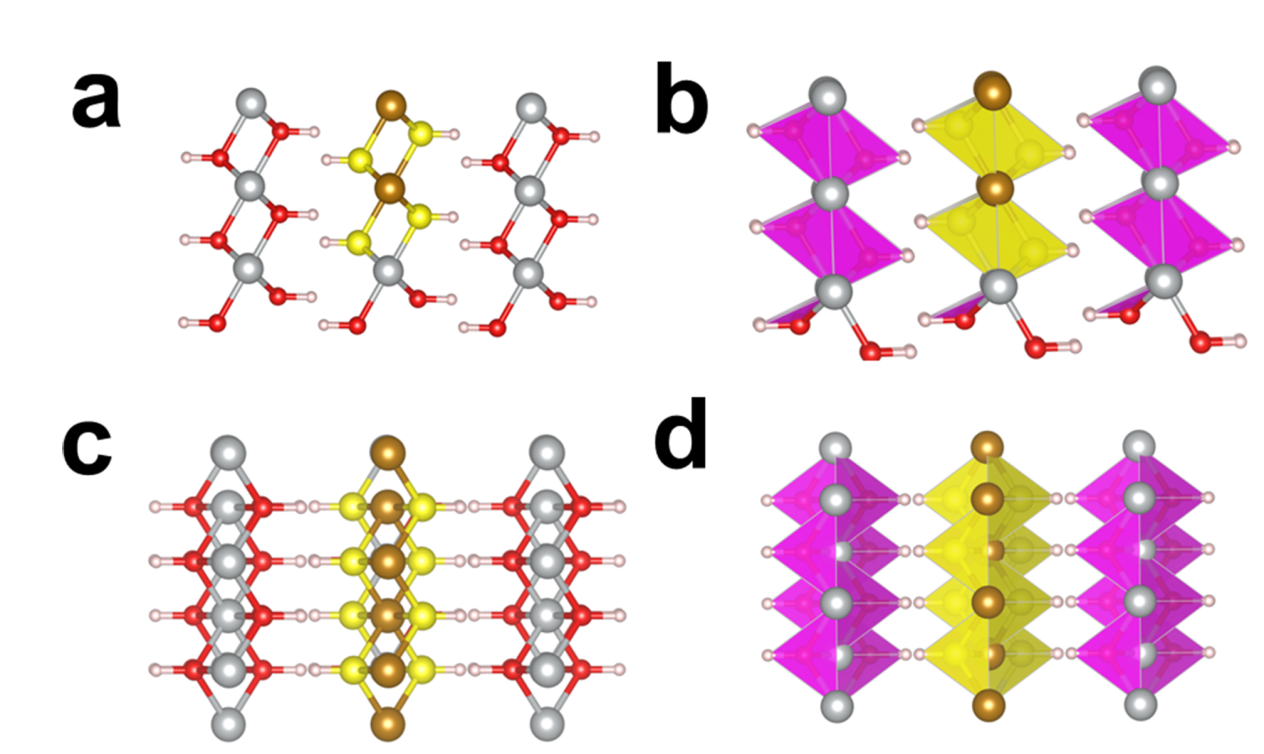
**

**Supplementary Figure 16│Structure models of Ni(Fe)(OH)_2_-Fe_4_S_12_.** Side view of ball-and-stick (a) and polyhedral model (b), top view of ball-and-stick (c) and polyhedral model (d).

**
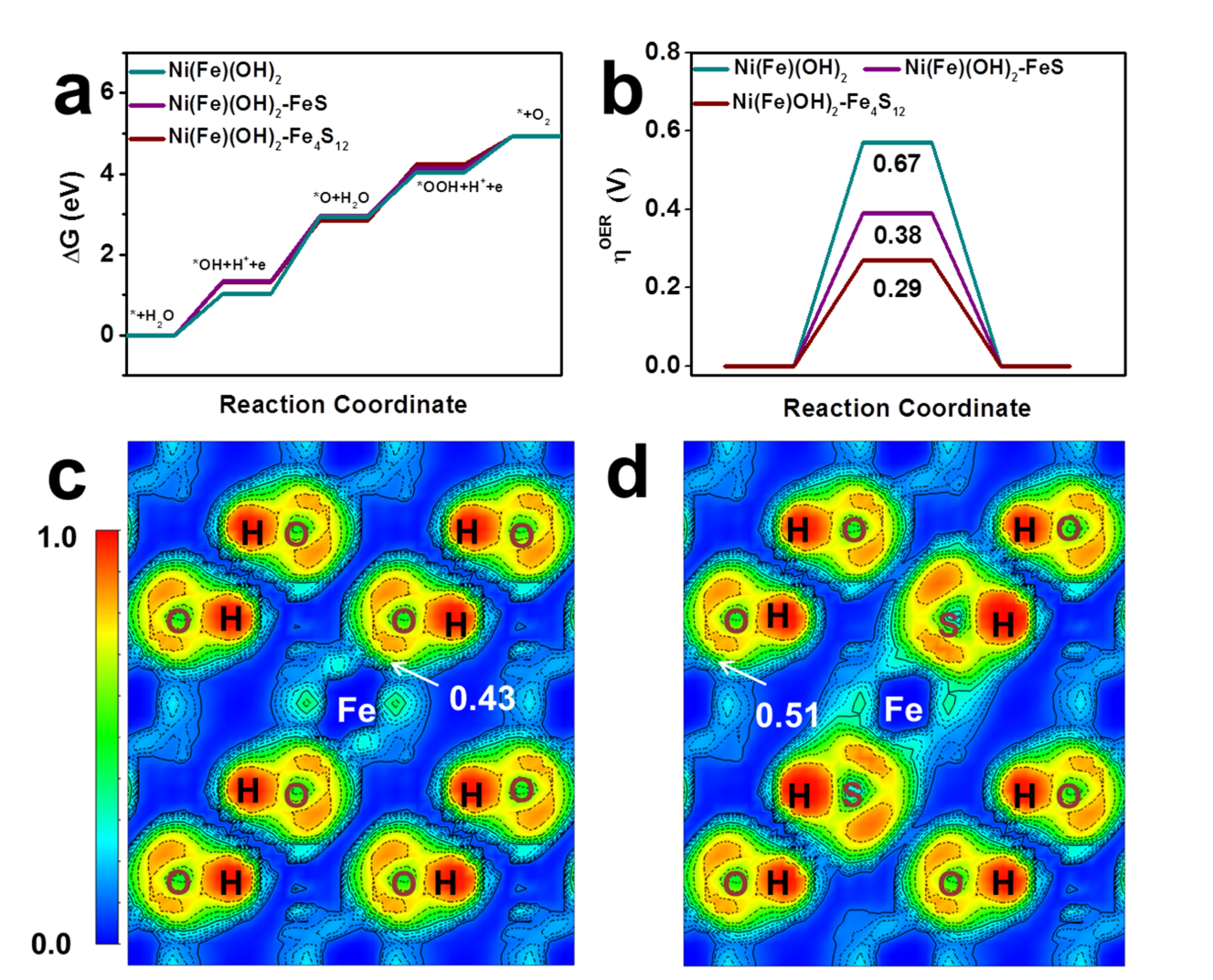
**

**Supplementary Figure 17│DFT calculations of** **different samples.** (a) Free energy diagram, (b) overpotential calculation of Ni(Fe)(OH)_2_, Ni(Fe)(OH)_2_-FeS and Ni(Fe)(OH)_2_-Fe_4_S_12_. Electron localization function of Ni(Fe)(OH)_2_ (c) and Ni(Fe)(OH)_2_-Fe_4_S_12_ (d).


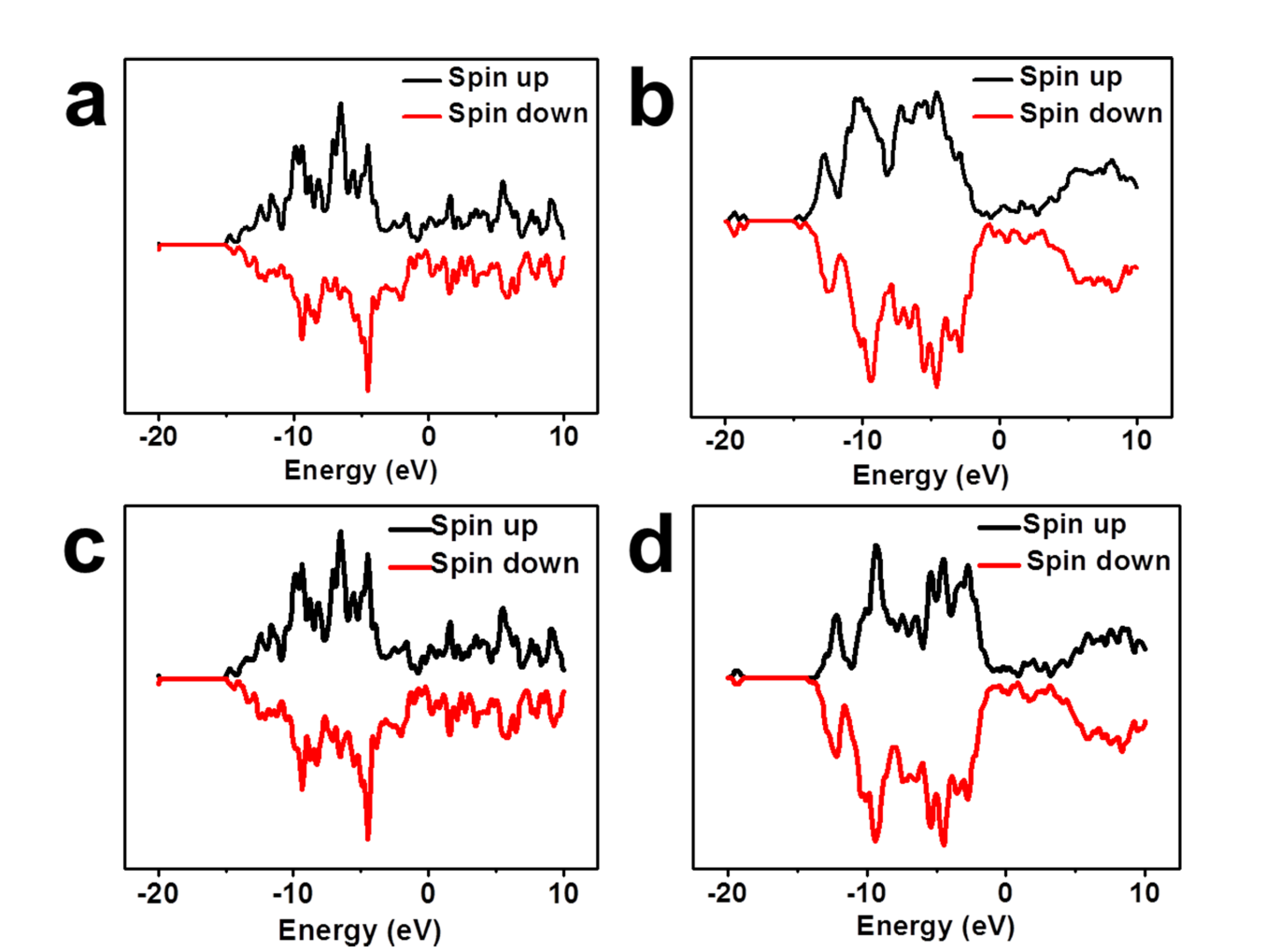


**Supplementary Figure 18│The calculated density of state of different samples.** (a) Ni(Fe)(OH)_2_, (b) Ni(Fe)OOH, (c) Ni(Fe)(OH)_2_-Fe_4_S_12_, and Ni(Fe)OOH-Fe_4_S_12_.

**Supplementary Tables**

**Supplementary Table 1│**The media of SRB.

| Medium  (g/L) | K_2_HPO_4_ | MgSO_4_·7H_2_O | (NH)_2_Fe(SO_4_)_2_ | NaCl | Yeast  extract | Vitamin  C | Sodium lactate (ml/L) | pH |
| --- | --- | --- | --- | --- | --- | --- | --- | --- |
| SRB | 0.01 | 0.2 | 0.2 | 10 | 1.0 | 0.1 | 4.0 | 7.2 |

**Supplementary Table 2│**EXAFS analysis results of the Ni(Fe)(OH)_2_-FeS_x_ and Ni(Fe)OOH-FeS_x_.

| Sample | Path | CN | R (Å) | σ^2^ (Å^2^) | ∆E_0_ (eV) | R-factor |
| --- | --- | --- | --- | --- | --- | --- |
| Ni(Fe)OOH-FeS_x_ | Fe-O | 2.9 | 1.91 | 0.0026 | -19.00 | 0.011 |
|  | Fe-S | 2.6 | 2.14 | 0.0180 | -19.41 |  |
|  | Fe-Ni | 4.7 | 3.10 | 0.0071 | -6.11 |  |
| Ni(Fe)(OH)_2_-FeS_x_ | Fe-O | 1.7 | 1.90 | 0.0000 | -11.54 | 0.020 |
|  | Fe-S | 1.0 | 2.18 | 0.0007 | -19.92 |  |
|  | Fe-Ni | 1.8 | 3.12 | 0.0032 | -0.45 |  |

^[a]^ CN = coordination number; ^[b]^ R = distance between absorber and backscatter atoms; ^[c]^ σ^2^= Debye-Waller factor

**Supplementary Table 3│**Performance comparison of recently reported OER electrodes in 1.0 M KOH solution.

| **Electrode** | **Preparation**  **method** | ***η* at 10 mA**  **cm^-2^ (mV)** | **Tafel slope**  **(mV dec^-1^)** | **Reference** |
| --- | --- | --- | --- | --- |
| NiFe/Graphene | Hydrothermal | 310 | 39 | *Sci. Adv.* **2018**, 4, 7970 |
| MoO_2_ Nanosheets | Wet-chemical and annealing | 260 | 54 | *Adv. Mater.* **2016**, 28, 3785 |
| Iron fluoride-oxide | Room-temperature synthesis | 270 | 45 | *Nat. Commun.* **2018**, 9, 1809 |
| NiCoO microcuboids | Self-assembled | 290 | 53 | *Angew. Chem*. **2016**, 128, 6398 |
| Fe-Mn-O Nanosheets | Reflux-Annealing | 273 | 64 | *Adv. Funct. Mater.* **2018**, 28, 1802463 |
| Ni-Bi@NB | Aqueous reaction | 302 | 52 | *Angew. Chem.* **2017**, 129, 6572 |
| NiCeO-Au | Electrodeposition | 270 | - | *Angew. Chem.* **2016**, 55, 3694 |
| NiCoP | Electrodeposition | 239 | 45 | *Adv. Funct. Mater.* **2016**, 26, 7644 |
| NiFe/Co_9_S_8_/carbon cloth | Hydrothermal-deposition | 219 | 55 | [*Nanoscale*](https://doi.org/10.1039/2040-3372/2009)*,* **2019***,* 11, 3378 |
| MoS_2_/Ni_3_S_2_ | Solvothermal | 218 | 88 | *Angew. Chem.* **2016**, 128, 6814 |
| Cu_1−x_NNi_3−y_/FeNiCu | Annealing | 300 | 52 | *Nat. Commun.* **2018**, 9, 2326 |
| FeNi@NC | Encapsulation | 280 | 70 | *Energy Environ. Sci.* **2016**, 9, 123 |
| NiFe/NF electrode | Electrodeposition | 270 | 28 | *Nat. Commun.* **2015**, 6, 6616 |
| FePO_4_/NF electrode | Two-phase colloidal | 218 | 43 | *Adv. Mater.* **2017**, 29, 1704574 |
| NiVIr-LDH | Hydrothermal | 180 | 38 | *Nat Commun.* **2019**, 10, 3899 |
| CoFe_0.25_Al_1.75_O_4_ | Sol-gel | 280 | - | *Nat Catal.* **2019**, 2, 763 |
| WCoFe oxyhydroxides | Facile wet-chemical | 250 | 32 | *Angew. Chem*. **2017**, 129, 4573 |
| Co-Zn oxyhydroxide | Co-precipitation | 235 | 36 | *Nat Energy.* **2019**, 4, 329 |
| NiFeV oxyhydroxide | Hydrothermal | 200 | 39 | *Nat. Commun.* **2018**, 9, 2885 |
| FeCoW oxyhydroxides | Room-temperature synthesis | 191 | - | *Science* **2016**, 352, 333 |
| CoFe-LDH nanosheet | Water-plasma exfoliation | 232 | 36 | *Adv. Mater.* **2017**, 29, 1701546 |
| Co-Fe Oxyphosphide | Hydrothermal | 280 | 53 | *Adv. Sci.* **2019**, 6, 1900576 |
| FeNi-GO LDH | Exfoliation and assembly | 210 | 40 | *Angew. Chem.* **2014**, 53, 7584 |
| Core-shell NiFeCu | Electrodeposition | 180 | 33 | *Nat. Commun.* **2018**, 9, 381 |
| NiFeCP/NF | Electrodeposition | 188 | 29 | *Nat. Commun.* **2019**, 10, 5074 |
| Au/NiFe LDH | Electrodeposition | 237 | 36 | *J. Am. Chem. Soc.* **2018**, 140, 3876 |
| NiFe LDH@NiCoP/NF | Hydrothermal-phosphorization | 220 | 48 | *Adv. Funct. Mater.* **2018**, 28, 1706847 |
| Ni(Fe)-MOF | Semisacrificial template | 227 | 39 | *Adv. Funct. Mater.* **2019**, 29, 1807418 |
| FeOOH(Se)/IF | Hydrothermal-oxidation | 287 | 54 | *J. Am. Chem. Soc.* **2019**, 141, 7005 |
| W-Ni(OH)_2_ | Alcohothermal method | 237 | 33 | *Nat. Commun.* **2019**, 10, 2149 |
| LaNiFe oxyhydroxide | Solvothermal | 189 | 36 | *Adv. Mater*. ***2019****,* 31, 1900883 |
| NiFe_2_O_4_/NiFe LDH | Hydrothermal | 180 | 28 | *ACS Appl. Mater. Interfaces* **2018**, 10, 26283 |
| NiFe-LDH | Hydrothermal method | 240 | - | *Science* **2014**, 345, 1593 |
| NiFe-LDH | Aqueous reaction | 230 | 47 | *Adv. Energy. Mater.* **2019**, 9, 1900881 |
| NiFe-LDH | Corrosion engineering | 269 | 48 | *Nat. Commun.* **2018**, 9, 2609 |
| NiFe-LDH | Electrodeposition | 215 | 28 | *Nat. Commun.* **2015**, 6, 6616 |
| NiFe-LDH | Room-temperature synthesis | 189 | 36 | *Adv. Mater.* **2019**, 31, 1900883 |
| NiFe-LDH | Hydrothermal | 184 | 34 | *Energy Environ. Sci.* **2019**, 12, 572 |
| **Ni(Fe)OOH-FeS_x_** | **Microorganism corrosion** | **220** | **55** | **This work** |

**Supplementary Table 4│**The free energy of different primitive reactions and *η*_OER_ of oxygen evolution for the calculated catalysts.

|  | ΔG_1_  (* → *OH) | ΔG_2_  (*OH → *O) | ΔG_3_  (*O → *OOH) | ΔG_4_  (*OOH → *) | *η*_OER_  (V) |
| --- | --- | --- | --- | --- | --- |

| NiFe(OH)_2_ | 1.04 | 1.9 | 1.09 | 0.89 | 0.67 |
| --- | --- | --- | --- | --- | --- |

| NiFe(OH)_2_-FeS | 1.35 | 1.61 | 1.19 | 0.77 | 0.38 |
| --- | --- | --- | --- | --- | --- |

| NiFe(OH)_2_-Fe_4_S_12_ | 1.33 | 1.52 | 1.38 | 0.69 | 0.29 |
| --- | --- | --- | --- | --- | --- |
| NiFeOOH | 1.5 | 1.68 | 1.14 | 0.6 | 0.45 |
| NiFeOOH-FeS | 1.37 | 1.44 | 1.08 | 1.03 | 0.21 |
| NiFeOOH-Fe_4_S_12_ | 1.34 | 1.37 | 1.25 | 0.96 | 0.14 |

**Supplementary Table 5│**The net charge at Fe atoms and Fermi level for the calculated catalysts.

|  | Net Charge at Fe atoms | Fermi level |
| --- | --- | --- |

| NiFe(OH)_2_ | 0.227 | -2.46 |
| --- | --- | --- |

| NiFeOOH | 0.243 | -2.72 |
| --- | --- | --- |

| NiFe(OH)_2_-Fe_4_S_12_ | 0.287 | -2.67 |
| --- | --- | --- |
| NiFeOOH-Fe_4_S_12_ | 0.496 | -2.88 |

**Supplementary References**

1. Friebel, D. et al. Identification of highly active Fe sites in (Ni,Fe)OOH for electrocatalytic water splitting. *J. Am. Chem. Soc.* **137**, 1305-1313 (2015).

2. Kresse, G. & Joubert, D. From ultrasoft pseudopotentials to the projector augmented-wave method. *Phys. Rev. B* **59**, 1758-1775 (1999).

3. Becke, A.D., and Kenneth E. Edgecombe. A simple measure of electron localization in atomic and molecular systems. *J. Chem. Phys.* **92**, 5397-5403 (1990).

4. Wu, S.Y. & Ho, J.J. Adsorption, dissociation, and hydrogenation of CO_2_ on WC(0001) and WC-Co alloy surfaces investigated with theoretical calculations. *J. Phys. Chem. C* **116**, 13202-13209 (2012).
