# Supplementary material for: Preparation of nickel-iron hydroxides by microorganism corrosion for efficient oxygen evolution
Source: Nat Commun. 2020 Oct 8;11:5075. doi: 10.1038/s41467-020-18891-x (PMC7545195; doi:10.1038/s41467-020-18891-x)
Supplement: Supplementary file 3 — Description of Additional Supplementary Files [file 41467_2020_18891_MOESM3_ESM.pdf]

Description of Additional Supplementary Files

File Name: Supplementary Movie 1

Description: The stability test of Ni(Fe)OOH-FeS_x electrode with area of 1×1 cm^2 at constant current density of 10 mA cm^{-2}.

File Name: Supplementary Movie 2

Description: The stability test of Ni(Fe)OOH-FeS_x electrode with area of 1×1 cm^2 at constant current density of 100 mA cm^{-2}.

File Name: Supplementary Movie 3

Description: The stability test of Ni(Fe)OOH-FeS_x electrode with area of 7×7 cm^2 at constant current density of 10 mA cm^{-2}.

File Name: Supplementary Movie 4

Description: The stability test of Ni(Fe)OOH-FeS_x electrode with area of 7×7 cm^2 at constant current density of 100 mA cm^{-2}.
