# Supplementary material for: Preparation of nickel-iron hydroxides by microorganism corrosion for efficient oxygen evolution
Source: Nat Commun. 2020 Oct 8;11:5075. doi: 10.1038/s41467-020-18891-x (PMC7545195; doi:10.1038/s41467-020-18891-x)
Supplement: Supplementary file 2 — Peer Review File [file 41467_2020_18891_MOESM2_ESM.pdf]

Editorial Note: This manuscript has been previously reviewed at another journal that is not operating a transparent peer review scheme. This document only contains reviewer comments and rebuttal letters for versions considered at *Nature Communications.*

Reviewer #1 (Remarks to the Author):

The authors have now significantly improved the study. They have clarified that S is in lower presence that the other elements with the changed notation to FeSx, added XAFS and analysis of effects on crystallinity. The lower presence of FeS after the activation is now accounted for at page 8 with "Compared with the initial Ni(Fe)(OH)2-FeSx, the decreased content of Fe-S and the enhanced content of S-O in the Ni(Fe)OOH-FeSx suggest some partially oxidized S species. Nevertheless, most Fe-S species are preserved in the Ni(Fe)OOH-FeSx." With this and the other clarifications, added analysis and more relevant DFT calculations, the study is now of high quality.

The only remaining concern is the new data presented in this revision at page 10 with " Remarkably, the SRB corroded Fe foam exhibits a greatly enhanced activity, which requires an η of only 175 mV to achieve the current density of 10 mA cm-2 (Supplementary Fig. S12a)."

As can be seen in supplementary Fig S12a, the 10 mAcm-2 current line where a voltage of 1.405 V vs RHE is extracted is at a place where the current contains a large portion of catalyst self-oxidation current. The scaling and guiding line of the catalytic load curve instead show that a realistic overpotential would be similar to that in Fig S12d, i.e. around 1.47V (giving an overpotential of 1.47V-1.23V=0.24 V or possibly slightly lower but definitely not 175 mV). A similar problem occur in Fig 12h. In supplementary Fig S13e and S13f, the authors correctly disregard the catalyst self-oxidation current and extract the behavior from the catalytic current and thus the correct overpotential. Since the self-oxidation current and the catalytic current are overlapping in the region, fitting of the load curve is needed or just a potential value at a higher current and using the Tafel-slope to estimate the overpotential at 10mAcm-2. From inspection of Fig S12a (and comparison with fig S12d and the other curves), the resulting overpotential would most likely be in the range 220-240 mV. The value of 175 mV needs to be updated with this value (without contribution from the catalyst self-oxidation current) in the aforementioned sentence at page 10, Fig S12a, and in the last row of supplementary Table S2. It is still clear that the corroded Fe has a lower overpotential than the blank Fe, but the champion overpotential value need to be corrected for the catalyst self-oxidation current.

In summary, a much-improved analysis and discussion and I recommend publication of the manuscript after the minor correction of the extracted overpotential at 10mAcm-2 for the corroded Fe at page 10, supplementary Fig S12a, and Table S2 as outlined above.

Reviewer #2 (Remarks to the Author):

The authors have done a fairly decent job at addressing properly the issues raised by the reviewer, and thus the reviewer is fine with the publication of this work in Nature Commun. There is though one minor suggestion: the insets (for structures) in Figure 6b are not properly positioned to correspond to the intermediates along the free energy profile, and why not draw the free energy profile at U = 1.23 V (vs SHE), which is the most common way and would show clearly the overpotential and also the potential-limiting step? If drawn in this manner, Figure 6c is not necessary (in fact it is a quite strange plot and seemingly implies that the OER has only one intermediate).

Reviewer #1:

The authors have now significantly improved the study. They have clarified that S is in lower presence that the other elements with the changed notation to FeS_x, added XAFS and analysis of effects on crystallinity. The lower presence of FeS after the activation is now accounted for at page 8 with "Compared with the initial $Ni(Fe)(OH)_2$-FeS_x, the decreased content of Fe-S and the enhanced content of S-O in the Ni(Fe)OOH-FeS_x suggest some partially oxidized S species. Nevertheless, most Fe-S species are preserved in the Ni(Fe)OOH-FeS_x." With this and the other clarifications, added analysis and more relevant DFT calculations, the study is now of high quality.

Our response: Thank you very much for your appreciation and encourage on our work. Based on your valuable comments and suggestions, we revised the manuscript carefully responses to the comments.

Comments: The only remaining concern is the new data presented in this revision at page 10 with " Remarkably, the SRB corroded Fe foam exhibits a greatly enhanced activity, which requires an η of only 175 mV to achieve the current density of 10 mA cm^{-2} (Supplementary Fig. S12a)."

As can be seen in supplementary Fig S12a, the 10 mA cm^{-2} current line where a voltage of 1.405 V vs RHE is extracted is at a place where the current contains a large portion of catalyst self-oxidation current. The scaling and guiding line of the catalytic load curve instead show that a realistic overpotential would be similar to that in Fig S12d, i.e. around 1.47 V (giving an overpotential of 1.47 V-1.23 V=0.24 V or possibly slightly lower but definitely not 175 mV). A similar problem occur in Fig 12h. In supplementary Fig S13e and S13f, the authors correctly disregard the catalyst self-oxidation current and extract the behavior from the catalytic current and thus the correct overpotential. Since the self-oxidation current and the catalytic current are overlapping in the region, fitting of the load curve is needed or just a potential value at a higher current and using the Tafel-slope to estimate the overpotential at 10 $mAcm^{-2}$. From inspection of Fig S12a (and comparison with fig S12d and the other curves), the resulting overpotential would most likely be in the range 220-240 mV. The value of 175 mV needs to be updated with this value (without contribution from the catalyst self-oxidation current) in the aforementioned sentence at page 10, Fig S12a, and in the last row of supplementary Table S2. It is still clear that the corroded Fe has a lower overpotential than the blank Fe, but the champion overpotential value need to be corrected for the catalyst self-oxidation current.

In summary, a much-improved analysis and discussion and I recommend publication of the manuscript after the minor correction of the extracted overpotential at 10 mA cm^{-2} for the corroded Fe at page 10, supplementary Fig S12a, and Table S2 as outlined above.

Our response: Thank you for your professional suggestions. We strongly agree with you that the self-oxidation current and the catalytic current are overlapped in the current density region of 10 mA cm^{-2} (Supplementary Fig. 12a, h and Fig. 13d, f). According to your good suggestions, a higher current (50 mA cm^{-2}) was adopted to avoid the self-oxidation current, and the extracted potential values were corrected in Supplementary Fig. 12a, h and Fig. 13d, f. Furthermore, based on your professional suggestions, we adopted potential value at a higher current and using the Tafel slope to estimate the overpotential at 10 mA cm^{-2}. As a results, the Tafel slope of the corroded Fe electrode is 52 mV dec^{-1} and the obtained overpotential at 10 mA cm^{-2} (η_{10}) is (~230 mV). Moreover, we conducted the stablity test of the SRB corroded Fe electrode in 1.0 M KOH electrolyte at constant current densities of 10 mA cm^{-2} and 100 mA cm^{-2} (Figure R1). The results indicate the corroded Fe electrode can function steadily for at least 20 h at constant current densities of 10 mA cm^{-2} at ~1.46 V and 100 mA cm^{-2} at ~1.52 V. The obtained η_{10} (~230 mV) is slightly higher but the η_{100} (~290 mV) is slightly lower than that of the SRB corroded Ni electrode (η_{10} = 220 mV and η_{100} = 300 mV). To avoid the confusion, we revised the corresponding sentence at page 10 and the last row in supplementary Table 2 of SRB corroded Fe electrode, and mainly focus on the SRB corroded Ni electrode. We have corrected and highlighted these modifications in the revised manuscript.

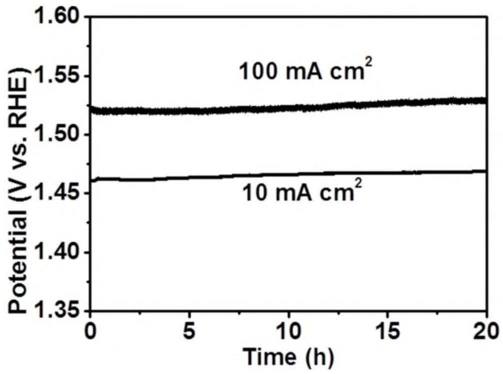

Figure R1. Chronopotentiometric curves obtained at constant current densities of 10 and 100 mA cm^{-2} of SRB corroded Fe.

Reviewer #2:

The authors have done a fairly decent job at addressing properly the issues raised by the reviewer, and thus the reviewer is fine with the publication of this work in Nature Commun.

Our response: Thank you very much for your appreciation and encourage on our work. Based on your valuable comments and suggestions, we revised the manuscript carefully responses to the comments.

Comments: There is though one minor suggestion: the insets (for structures) in Figure 6b are not properly positioned to correspond to the intermediates along the free energy profile, and why not draw the free energy profile at U = 1.23 V (vs SHE), which is the most common way and would show clearly the overpotential and also the potential-limiting step? If drawn in this manner, Figure 6c is not necessary (in fact it is a quite strange plot and seemingly implies that the OER has only one intermediate).

Our response: Thank you for your professional comments. We modified the insets (for structures) in Figure 6b with proper position corresponded to the intermediates along the free energy profile. We strongly agree with you that the free energy profile at U = 1.23 V (vs SHE) can show the overpotential and also the potential-limiting step, and we also draw the free energy profile at U = 1.23 V (vs SHE) in Figure 6b. We used Figure 6c to visually demonstrate the overpotential of the rate-determining step (RDS). According to your valuable suggestions, we removed the Figure 6c and added the corresponding overpotential of RDS in Supplementary Table 4. We have modified and highlighted them in the revised manuscript.

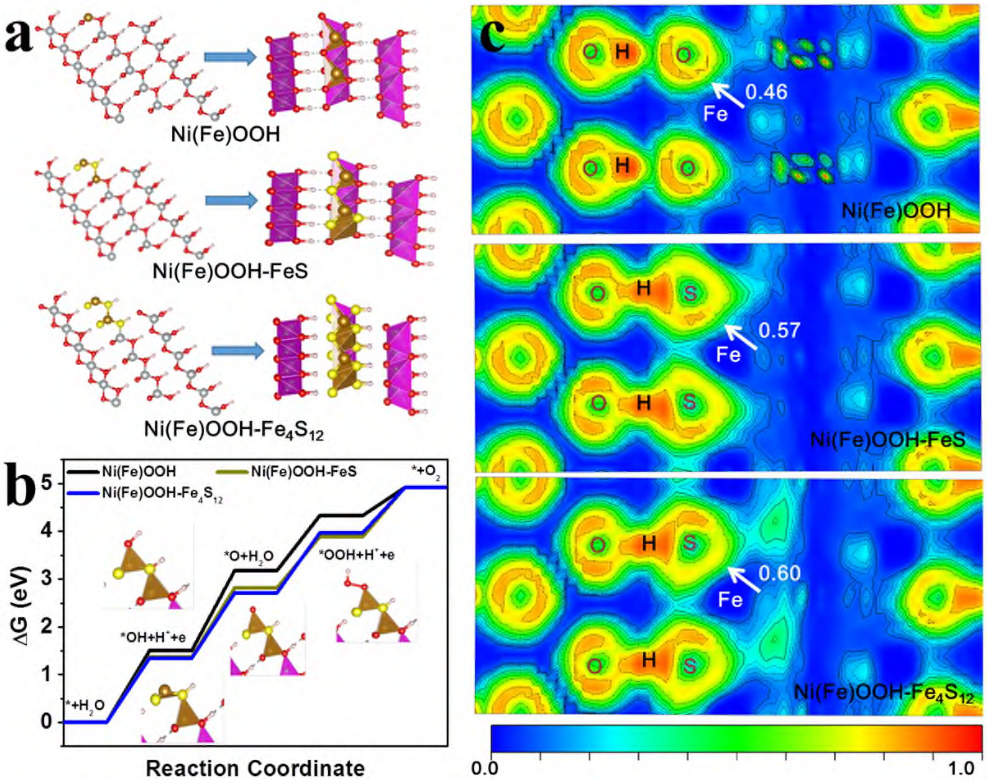

Figure 6. DFT calculations of different electrodes. (a) Structural model, (b) free energy diagram, (c) electron localization function.

Supplementary Table 4 | The free energy of different primitive reactions and η_{OER} of oxygen evolution for the calculated catalysts.

	ΔG_1 (* → *OH)	ΔG_2 (*OH → *O)	ΔG_3 (*O → *OOH)	ΔG_4 (*OOH → *)	η_{OER} (V)
$NiFe(OH)_2$	1.04	1.9	1.09	0.89	0.67
$NiFe(OH)_2$-FeS	1.35	1.61	1.19	0.77	0.38
$NiFe(OH)_2$-Fe_4S_{12}	1.33	1.52	1.38	0.69	0.29
NiFeOOH	1.5	1.68	1.14	0.6	0.45
NiFeOOH-FeS	1.37	1.44	1.08	1.03	0.21
NiFeOOH-Fe_4S_{12}	1.34	1.37	1.25	0.96	0.14
